# The fungal gut microbiota in pediatric-onset multiple sclerosis

**DOI:** 10.3389/fmicb.2024.1258978

**Published:** 2024-12-18

**Authors:** Nelson Mok, Natalie C. Knox, Feng Zhu, Douglas L. Arnold, Amit Bar-Or, Charles Noah Bernstein, Christine Bonner, Jessica D. Forbes, Morag Graham, Ruth Ann Marrie, Julia O’Mahony, E. Ann Yeh, Yinshan Zhao, Gary Van Domselaar, Brenda Banwell, Emmanuelle Waubant, Helen L. Tremlett

**Affiliations:** ^1^Department of Medical Microbiology and Infectious Diseases, University of Manitoba, Winnipeg, MB, Canada; ^2^Science Technologies Operations, Public Health Agency of Canada, Winnipeg, MB, Canada; ^3^Department of Medicine, University of British Columbia, Vancouver, BC, Canada; ^4^Department of Neurology and Neurosurgery, McGill University, Montreal, QC, Canada; ^5^Department of Neurology, University of Pennsylvania, Philadelphia, PA, United States; ^6^Department of Internal Medicine, University of Manitoba, Winnipeg, MB, Canada; ^7^Eastern Ontario Regional Laboratory Association, Ottawa, ON, Canada; ^8^Department of Pediatrics, Division of Neurology, The Hospital for Sick Children, University of Toronto, Toronto, ON, Canada; ^9^Children’s Hospital of Philadelphia, Philadelphia, PA, United States; ^10^UCSF Weill Institute for Neurosciences, University of California, San Francisco, San Francisco, CA, United States

**Keywords:** multiple sclerosis, monophasic acquired demyelinating syndrome, pediatric, gut mycobiota, fungal gut microbiome, case–control

## Abstract

Evidence suggests that the gut microbiome may play a role in multiple sclerosis (MS). However, the majority of the studies have focused on gut bacterial communities; none have examined the fungal microbiota (mycobiota) in persons with pediatric-onset multiple sclerosis (POMS). We examined the gut mycobiota in persons with and without POMS through a cross-sectional examination of the gut mycobiota from 46 participants’ stool samples (three groups: 18 POMS, 13 acquired monophasic demyelinating syndromes [monoADS], and 15 unaffected controls). Using metataxonomic sequencing of the fungal internal transcribed spacer region 2, the fungal profiles were compared between participants using visualizations, statistical tests, and predictive analysis. While the mycobiome *α*- (Shannon and inverse Simpson indices) and *β*-diversity differed across the three groups [analysis of variance (ANOVA), *p* < 0.05], further *post-hoc* analysis of the *β*-diversity identified a difference between monoADS vs. POMS participants [*p* = 0.005 (adjusted)]. At the genus level of taxonomy, 7 out of 10 of the majority of abundant genera were similar among all three groups, with *Saccharomyces* spp. and *Candida* spp. being in the highest abundance. The *Agaricus* genus was especially high in POMS participants, dominated primarily due to the species *Agaricus bisporus* (widely consumed as white button mushrooms). The commonality of high abundance fungi found in our cohort suggests a possible connection to diet. Predictive modeling of differential abundance associated with *Candida albicans*, *Cyberlindera jadinii*, and *Fusarium poae* revealed that these fungi were strongly associated with the POMS participants. Our study provides novel insight into the fungal gut mycobiota in POMS. While findings indicate that the gut mycobiome of participants with POMS may largely comprise fungi considered transient from the diet, the differential predictive analysis suggested rare or under-detected fungal markers being of potential importance, warranting consideration in future mycobiome-MS-related studies.

## 1 Introduction

Multiple sclerosis (MS) is a complex, immune-mediated, and neurodegenerative disease of the central nervous system (CNS; Filippi et al., 2018) The cause(s) of MS remain incompletely understood, but emerging evidence suggests that the gut microbiota may play a role. Potential mechanisms are thought to include alterations in gut–brain signaling and disrupting tight junctions of the blood–gut and blood–brain barriers (Parodi and Kerlero de Rosbo, 2021; Cantoni et al., 2022).

The fungal component of the gut microbiota (the mycobiota) is often understudied, and while some evidence of a fungal etiology in MS exists; studies are limited. For example, the presence of fungi in the cerebrospinal fluid was reported in a case series of 12 MS participants using molecular and immunological assays (Pisa et al., 2013). Molecular signatures for fungi were also observed in the postmortem brain tissues of 8 of 10 MS participants when amplifying for the fungal internal transcribed spacer (ITS) region (Alonso et al., 2018). In mice with experimental autoimmune encephalomyelitis, a commonly used animal model for MS, an inoculation of the fungi *Candida albicans* exacerbated symptoms, and tissue analysis by histopathology of biopsy samples detected CNS fungal invasion (Fraga-Silva et al., 2015). Interestingly, the disease-modifying drug dimethyl fumarate is known to have antifungal effects and is approved for regulatory use to manage MS, although it is unclear whether its fungicidal effects are relevant in MS (Ma et al., 2017; Derfuss et al., 2020).

Investigation of the gut fungal role in MS is also lacking; to date, only two studies have reported on the association of the gut mycobiome and MS (Shah et al., 2021; Yadav et al., 2022). Both studies included adults and comprised 20–25 MS cases and 22–33 unaffected controls. To our knowledge, no study has investigated the role of the gut mycobiome in pediatric-onset multiple sclerosis (POMS) participants. Although POMS is considered a rare disease, it offers a unique opportunity to examine the gut mycobiome relatively early in the disease course, before the accrual of complex exposures such as comorbidities, medications, or tobacco use over several decades (MSIF, 2020; Yusuf et al., 2023).

We sought to investigate the gut fungal profiles in a cohort of POMS participants and compared their gut fungal profiles to participants with a monophasic acquired demyelinating syndrome (monoADS) and to unaffected participants using stool samples. The findings reported here address the knowledge gap regarding the existence of a gut fungal association between POMS and unaffected controls.

## 2 Materials and methods

### 2.1 Study cohort

Our study cohort included participants enrolled in a Canadian Pediatric Demyelinating Disease Network study, details of which have been described previously (Tremlett et al., 2021). Briefly, to be included in the current study, participants had to be ≤24 years of age at the time of stool sample collection (between 2015 and 2019), and have provided the stool sample without taking an antibiotic in the prior 30 days. Participants included were those diagnosed with POMS or monoADS with symptom onset (first clinical attack), <18 years of age, or who were unaffected controls. MS cases fulfilled the McDonald diagnostic criteria (Polman et al., 2011; Thompson et al., 2018). MonoADS was defined as an initial acute clinical episode of symptoms involving the CNS, with evidence of inflammatory demyelination and with no new/subsequent clinical or MRI findings of recurrent demyelination (Fadda et al., 2018). Unaffected controls had no known neurological or (auto)immune-related condition (headache/migraine, asthma, and allergies were permissible).

### 2.2 DNA extraction and sequencing from stool

Stool samples were shipped on ice and stored at −80°C before genomic DNA extraction using the Zymo Quick-DNA™ Fecal/Soil Microbe Miniprep Kit (Zymo Research, Irvine, CA, USA). Genomic DNA library preparation targeting the fungal internal transcribed spacer 2 (ITS2) region was PCR amplified using forward primer ITS7-XT99 (TCGTCGGCAGCGTCAGATGTGTATAAGAGACAGGTGARTCATCGAATCTTTG) and reverse ITS4-XT101 (GTCTCGTGGGCTCGGAGATGTGTATAAGAGACAGTCCTCCGCTTATTGATATGC), where non-underlined nucleotides correspond to Illumina overhang adapter sequences. The targeted amplicon polymerase chain reaction (PCR) reaction was performed using the KAPA HiFi HotStart ReadyMix (Roche, Pleasanton, CA, USA). The resulting ITS2 amplicons were then purified with 20-μl AMPure XP (Beckman Coulter Canada, LP, Mississauga, Ontario, Canada), and a secondary amplification was performed to attach multiplexing indices. Indexed amplicons were purified using 56-μl AMPure XP, and then the libraries with positive concentrations were quantitated using PicoGreen and pooled in equimolar amounts. Gating of the pooled libraries was selected using BluePippin 1.5% cassettes (Sage Science, Inc., Beverly, MA, USA) for 300–1,000 bp fragments. Size-selected libraries were then purified using 0.6X AMPure XP, assessed for size on an Agilent Tapestation analyzer (Agilent Technologies Canada, Inc., Mississauga, Ontario, Canada), and quantitated on a Qubit 2.0 (Thermo Fisher Scientific, Inc., Waltham, MA, USA).

The libraries were then denatured using 0.5 N NaOH to 11 pM and spiked with 25% PhiX control DNA and subsequently sequenced using the Illumina, San Diego, CA, USA V3 (600 cycles; 2 × 300 bp). A total of 134 samples were sequenced, 65 in the first run and 69 in the second. Each MiSeq run was performed with an extraction water blank no-template control, and a positive control (mock community). A nineteen-taxon mock fungal community of “Staggered A” 18S rRNA proportions was used in duplicate as a positive control (Bakker, 2018). The resulting FASTQ sequences were demultiplexed and assessed for sequencing quality by fast quality control (FastQC - Bioinformatics pipeline is open source but developed at: Babraham Institute, Cambridge, UK) v0.11.9 and multiple quality control (MultiQC-Bioinformatics pipeline is open source but developed at: National Genomics Infrastructure, Stockholm, Sweden) v1.8 (Andrews, 2010; Ewels et al., 2016).

### 2.3 Sequence clustering and taxonomic assignment

Sequences of fungal ITS2 amplicons from Illumina MiSeq were processed using the less operational taxonomic unit script (LotuS)(Bioinformatics pipeline is open source but developed at: Quadram Institute Bioscience (QIB) & Earlham Institaute (EI), Norwich, UK) and sdm v1.62 pipeline (Hildebrand et al., 2014). The *lotus.pl* script was run using the following parameters: gate sequences of length 100–1,000 bp, post-trimming minimum average base quality of 33, removal of sequences with ambiguous bases, maximum homopolymer length of 15 bp, base quality of ≥25 within a 50-bp k-mer window, and 50 bp sequence ends were trimmed if the sequence quality score fell below 25.

The Unified Search (USEARCH) v11.0.667 sequence clustering tool was used to cluster reads with a ≥ 97% sequence identity (Edgar, 2010). Chimeric and PhiX sequences were removed by alignment against the Unoise CHIMEras (UCHIME) v7.2 fungal database (Nilsson et al., 2019b). Read pairs overlapping by at least 10 bp with an overall average quality score of 33 were merged using Fast Length Adjustment of SHort Reads (FLASH: John Hopkins University, Baltimore, MD USA) v1.2.10 (Magoč and Salzberg, 2011). The merged reads were then subjected to filtering for fungal ITS2 sequences by the ITSx tool: Chalmers University of Technology and University of Gothenburg, Goteborg, Sweden (Bengtsson-Palme et al., 2013). Fungal taxonomic assignment was designated by alignment against the mothur release of the UNITE v8.2 fungal ribosomal database using the Basic Local Alignment Search Tool+ (BLAST+: National Institutes of Health, Bethesda, MD, USA Camacho et al., 2009; Nilsson et al., 2019b). Standard parameters were used for all bioinformatics tools of this analysis when applicable.

### 2.4 Data cleaning, counts transformation, and mock community checks

The R package *LULU* was used to group operational taxonomic units (OTUs) with a high sequence similarity in an attempt to decrease spurious counts without removing them from downstream statistical analysis (Frøslev et al., 2017). A matched list of sequences is used to align the fungal ITS2 identities characterized by the LotuS pipeline against itself in an all-against-all similarity comparison using BLAST+. The parameters set for BLAST+ retained sequence alignments with ≥84% sequence identity and ≥ 80% query coverage per high-scoring sequence pair. Good’s coverage index was calculated for each sample in the resulting table of read counts, and samples with an overall count of ≤500 OTUs were excluded from the study to retain a coverage index of ≥96.5% (Good, 1953; Kowalchuk et al., 2004).

Since OTUs with zero counts cannot undergo a log transformation, the table of counts was transposed, and values with an abundance of 0 were given a pseudocount using the Geometric Bayesian multiplicative method with a threshold of 0.5 implemented by the *zCompositions* R package (Palarea-Albaladejo and Martín-Fernández, 2015). The table of OTU counts was then transformed using the center log ratio transformation via the *clr* function from the *compositions* R package (van den Boogaart et al., 2021). Central log ratio (CLR) transformed samples were visually inspected for normality and skewness by quantile–quantile (Q–Q) plot and density distribution using the *ggplot2* R package (Wickham, 2016). The mock community control was not subject to LULU curation or transformation of counts as the relative ratio and identities of the control are known. Instead, the mock community data was visually inspected for the presence or absence of expected taxa at the species level.

### 2.5 Top 10 genera and statistical analysis

Visual inspection of the top 10 genera was performed for participant groups (POMS, monoADS, or unaffected controls). To ease interpretation, raw counts were transformed to a proportion, while CLR-transformed values were used in the analyses (except for *α*-diversity). All visualizations were generated using the *ggplot2* R package (Wickham, 2016).

Biodiversity was estimated by α-diversity using the number of observed OTUs, and Shannon and inverse Simpson indices. α-Diversity was compared among the POMS, monoADS, and unaffected participants using the Kruskal–Wallis ANOVA and Dunn’s *post-hoc* testing using Benjamini–Hochberg correction via the *ggpubr* and *rstatix* R packages (Kassambara, 2020, 2021). As a complementary approach, α-diversity was also examined by sex or age at stool samples collection (grouped as “child” ≤14 years of age or “youth” 15–24 years of age) for all participants combined due to an insufficient number of study participants to perform statistical testing on groupings further stratified by diagnoses. The examination of α-diversity on sex or age group was performed using the Wilcoxon rank-sum test.

*β*-Diversity was examined using principal component analysis (PCA) and non-metric multidimensional scaling (NMDS) specifying the Euclidean distance with the aid of the *PCAtools* and *vegan* R packages (Blighe and Lun, 2020; Oksanen et al., 2020). Euclidean distances resulting from PCA and NMDS were compared between groups using permutational multivariate ANOVA (PERMANOVA), facilitated by the *adonis2* function of the *vegan* R package, followed by *pairwiseAdonis* (Arbizu, 2017). Pairwise *p*-values were adjusted by Benjamini–Hochberg correction using the *stats* R package (RCT, 2021).

Analysis of differential species abundance among the POMS, monoADS, and unaffected control participants was performed using the ANOVA-Like Differential Expression (*ALDEx2*) R package on untransformed raw counts and by linear discriminant analysis effect size (*LEfSe*: Harvard T.H. Chan, Boston, MA, USA) on CLR-transformed counts via the microbiomeMarker R package (Segata et al., 2011; Gloor et al., 2016; Cao, 2021). Both *ALDEx2* and *LEfSe* are statistical packages used for differential abundance analysis of biological data through predictions by machine learning-like steps. *ALDEx2* differs from *LEfSe* in its first step, whereby posterior probabilities are generated for the counts of each OTU, and Monte–Carlo sampling from a Dirichlet distribution is used to resample the data, which is then transformed (Fernandes et al., 2014). The *LEfSe* algorithm forgoes the resampling process and directly transforms OTU data in preparation for downstream steps. Both *ALDEx2* and *LEfSe* compare the POMS, monoADS, and unaffected control participants using the Kruskal–Wallis ANOVA and Wilcoxon rank sum test. Effect sizes (generated by *LEfSe*) were plotted using a modified *plot_ef_bar* function to display data according to taxonomic levels. In all statistical tests, an *α* level of ≤0.05 was used to indicate significance.

The BLAST+ sequence alignment software package and the BLAST fungi National Center for Biotechnology Information (NCBI) Reference Sequence (RefSeq) ITS database were used to reassign organisms resulting from the differential abundance analyses above the species level to a lower taxonomic level. The following BLAST+ parameters were used: *qcov_hsp_perc* 80 and *perc_identity* 80 (Schoch et al., 2014). The BLAST results were filtered for hits with the lowest expected value and the highest query coverage per high-scoring segment pair, followed by the highest percent identity.

To identify a larger number of *Unknown* and *unclassified Eurotiales* genera—which were particularly apparent in the monoADS participants (using the UNITE v8.2 fungal ribosomal database in the current study)—the latest update of the UNITE v10.0 (2024-04-04) was used in a local BLAST+ sequence alignment to provide possible reclassification of these organisms. The same parameters, as aforementioned, were used, but an additional filter for ≥97% percent identity was performed.

## 3 Results

### 3.1 Filtering and cohort characteristics

Of the 91 participants who provided a stool sample, following LULU curation and determination of Good’s coverage, 45 participants were removed due to low read counts (≤ 500), leaving 46 participants in the final analyses. Of those included, 18 had POMS, 13 had monoADS, and 15 were unaffected controls. Cohort characteristics are shown in Table 1. The mean age at symptom onset was younger for the monoADS participants than for the MS cases. The monoADS participants were also younger on average at stool sample collection than either the MS or unaffected participants. Fourteen of the 18 MS participants were exposed to a disease-modifying therapy at the time of stool sample collection or within the previous 90 days.

**Table 1:** Characteristics of the pediatric-onset multiple sclerosis (POMS) cases, monophasic acquired demyelinating syndrome (monoADS), and unaffected control participants.

Characteristics	POMS^a^^,^^b^ *n* = 18	MonoADS^c^ *n* = 13	Unaffected control *n* = 15
Sex, female: *n* (%)	15 (83%)	6 (46%)	8 (53%)
Sex, male: *n* (%)	3 (17%)	7 (54%)	7 (47%)
Age at symptom onset, years: mean (SD)	15.3 (2.8)	5.2 (3.2)	NA
Age at stool sample collection, years: mean (SD)	18.1 (2.4)	12.1 (3.6)	15.3 (3.4)
Disease-modifying drug exposure status at stool sample collection (ever): No (%)	14 (78)	NA	NA

^aPOMS, pediatric-onset multiple sclerosis.bOf those exposed to a disease-modifying drug, seven participants had used an IFNB, three glatiramer acetate, two dimethyl fumarate, one natalizumab, and one Rituximab.cmonoADS, monophasic acquired demyelinating syndrome. NA, not applicable; SD, standard deviation.^

### 3.2 Data processing

An average of 30,791,070 raw paired-end reads from the 46 participants were generated, while the mock community yielded, on average, 10,168,535 raw paired-end reads between technical replicates. After sequencing processing, filtering for quality and minimum sample size, and clustering, the 46 participants totaled 10,525,102 reads and 2,175,124 reads across mock community replicates (Table 2). Visual inspection of the density distribution for the number of reads in each participant stool community showed non-normal distribution, which was confirmed using a Q-Q plot (Supplementary Figures S1A,B), but following central log ratio (CLR) transformation, sample density profiles showed a nearly normal or right-skewed distribution (Supplementary Figures S2A,B). A check of CLR-transformed values using a Q–Q plot showed that the normality assumption was not met in the majority of the sequenced samples (Supplementary Figure S3).

**Table 2:** The number of reads for the 46 included participants (pediatric-onset multiple sclerosis cases, monophasic acquired demyelinating syndrome, and unaffected controls combined) following sequencing and bioinformatics processing.

	Raw sequencing (averaged)	Lotus^a^	LULU^b^
Total reads	30,791,070	10,525,102	10,500,725
Minimum	1,644	563	562^c^
Medium	11,3,768	37,945	37,907
Mean	669,371	228,807	228,277
Maximum	4,854,072	2,566,757	2,560,308

^aLess operational taxonomic unit scripts sequence processing pipeline.^

^b^LULU postclustering sequence filtration; includes filtering >500 reads (according to good’s coverage ≥96.5%).

^cThe resulting minimum is lower for LULU because a single OTU found in the previous steps was identified to be of non-fungal origins, and is therefore removed.^

**Figure 1:**
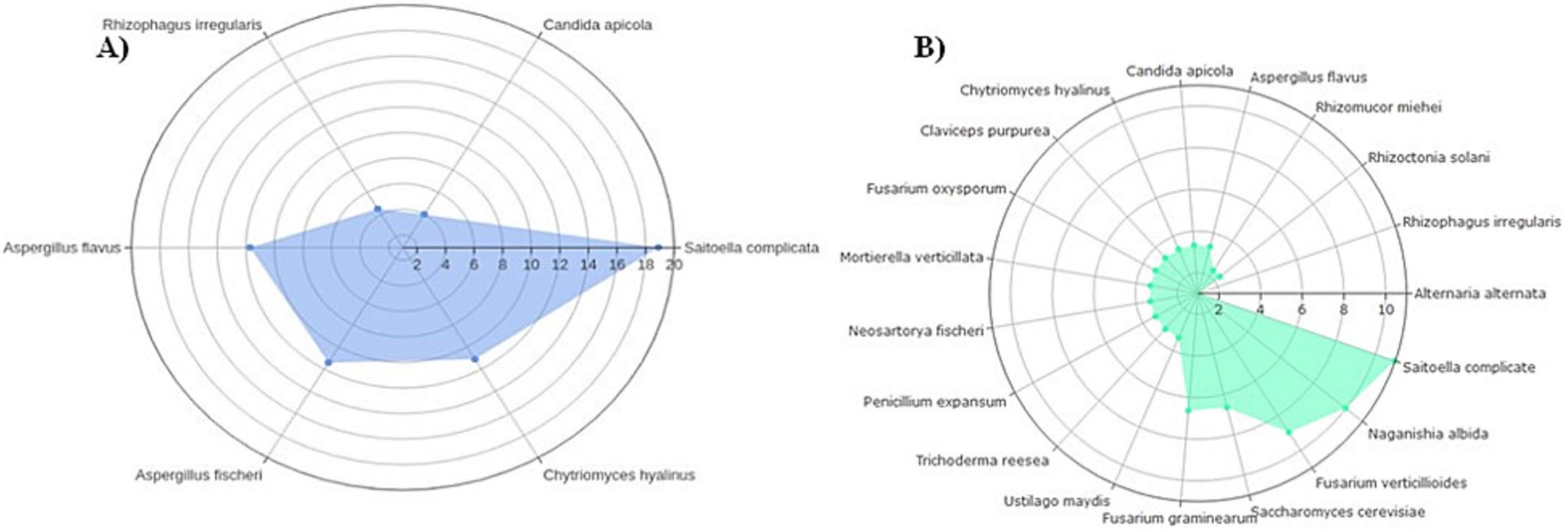
Relative ratios of a “Staggered A” 19-member mock fungal community transformed on a log2 scale used as study controls. In **(A)** fungal ITS2 ratios from this study averaged between technical replicates of six detected mock organisms; **(B)** expected 18S ribosomal RNS (rRNA) ratios of all 19 organisms (Bakker, 2018).

Out of 19 expected fungal organisms in the mock community, only six species were identified at the species level: *Saitoella complicata*, *Aspergillus fischeri* (also known as *Neosartorya fischeri—*the telomorphic form), *Aspergillus flavus*, *Chytriomyces hyalinus*, *Rhizophagus irregularis*, and *Candida apicola* (Figure 1A). The order of fungal abundance was in agreement with the “Staggered A” community generated in a previous study by Bakker, 2018, with the exception of *C. apicola*, which was found at a lower average abundance relative to *R. irregularis* due to under-sequencing/detection in the second replicate (Figure 1B).

### 3.3 Fungal taxon abundance

Descriptively, the Ascomycota phylum dominated across all three groups, representing 93.2% of the relative abundance for the POMS participants, 83.7% for the monoADS participants, and 95.4% for the unaffected controls, while Basidiomycota appeared higher for the POMS participants at 6.62% compared to monoADS (0.622%) and the unaffected controls (0.465%; Supplementary Figure S4). In contrast, Mucoromycota appeared lower in abundance for the POMS participants (0.0659%) compared to monoADS (1.38%) and unaffected controls (3.80%).

**Figure 2:**
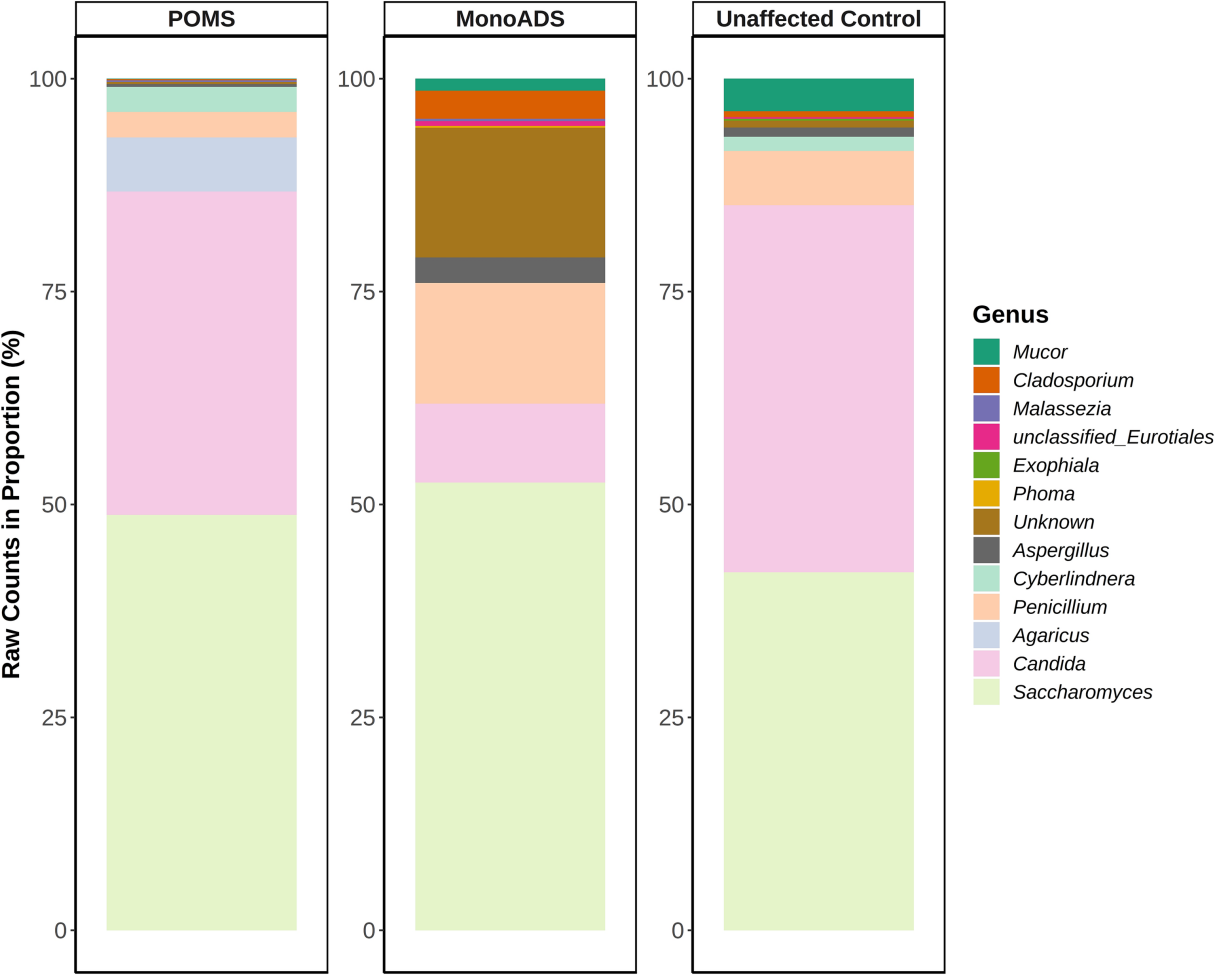
Top 10 most abundant fungal genera for pediatric-onset multiple sclerosis, monophasic acquired demyelinating syndrome, and unaffected control participants. Raw read counts are converted to a proportion in the percentage of the top genera.

Figure 2 shows the top 10 genera for the POMS, monoADS, and unaffected participants. The relative abundances of observed taxa are similar across the three groups, with *Saccharomyces* detected at the highest proportion, comprising 48.8% of the top 10 genera for the POMS participants, 52.6% for the monoADS, and 42.0% for the unaffected controls. Another six genera were shared among all three groups: *Aspergillus*, *Candida*, *Cladosporium*, *Mucor*, *Penicillium*, and an unidentified genus. The *Candida* genus was generally the second-highest in terms of relative abundance (38.0% for POMS, 9.3% monoADS, and 43.1% for unaffected control). Of the top 10 most abundant genera shared between two or more groups, only the genus *Cyberlindnera* was found for both the POMS and unaffected participants, while the genus *Malassezia* was found for the POMS and monoADS participants. In addition, an *unclassified* genus from the order Eurotiales was common to both the monoADS and unaffected participants. The genera *Aspergillus*, *Candida*, *Cladosporium*, *Mucor*, *Penicillium*, *Saccharomyces*, and unclassified *Eurotiales* were found in all three groups. Within the top 10 genera, *Agaricus* was found to be uniquely abundant in POMS participants, *Phoma* in monoADS, and *Exophiala* in unaffected controls. We also found that 99.9% of *Agaricus* species present for the POMS participants were composed of *A. bisporus*, and the remaining percentages belonged to an unclassified *Agaricus* spp. and *Agaricus kriegeri*. Although the *Agaricus* genus was not within the top 10 most abundant for the monoADS and unaffected controls, the species *A. bisporus* constituted 100% of this genus in monoADS and 89.8% in unaffected control participants.

### 3.4 *α*- and *β*-diversity by disease status

**Figure 3:**
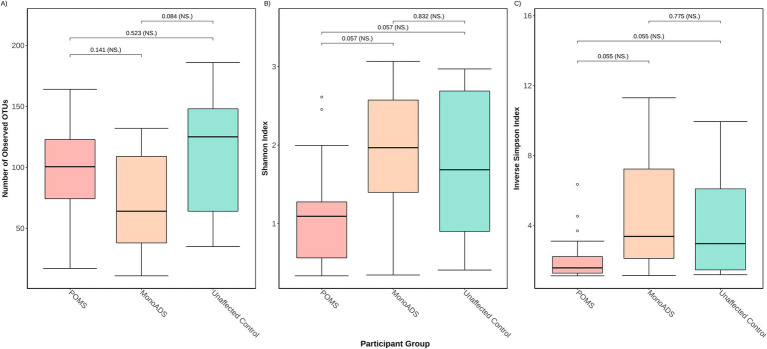
Three different *α*-diversity metrics were calculated for the pediatric-onset multiple sclerosis cases, monophasic acquired demyelinating syndrome, and unaffected control participants. In **(A)** the number of observed operational taxonomic units (OTUs) represents unique species—also known as the richness; **(B)** the Shannon diversity index accounts for richness and abundance but weighs more toward richness; **(C)** the inverse Simpson diversity index accounts for richness and abundances but weighs more toward abundance. Calculations are based on raw counts.

The α-diversity results are presented in Figures 3A–C. There were no statistically significant differences among the three groups for the number of unique species (*p* = 0.08 Kruskal–Wallis ANOVA and all *p* > 0.05 for the pairwise group comparisons [adjusted], Figure 3A). While the three groups differed for Shannon and inverse Simpson indices (*p* = 0.041) and Kruskal–Wallis ANOVA (*p* = 0.034), respectively (Figures 3B,C), none of the pairwise group comparisons reached significance after *post-hoc* testing (all *p* > 0.05 [adjusted]). Regarding the ratio of unique species, unaffected control participants exhibited higher gut mycobiota diversity than POMS (4:5 POMS:unaffected controls) and monoADS individuals (2.6:5 monoADS:unaffected controls).

**Figure 4:**
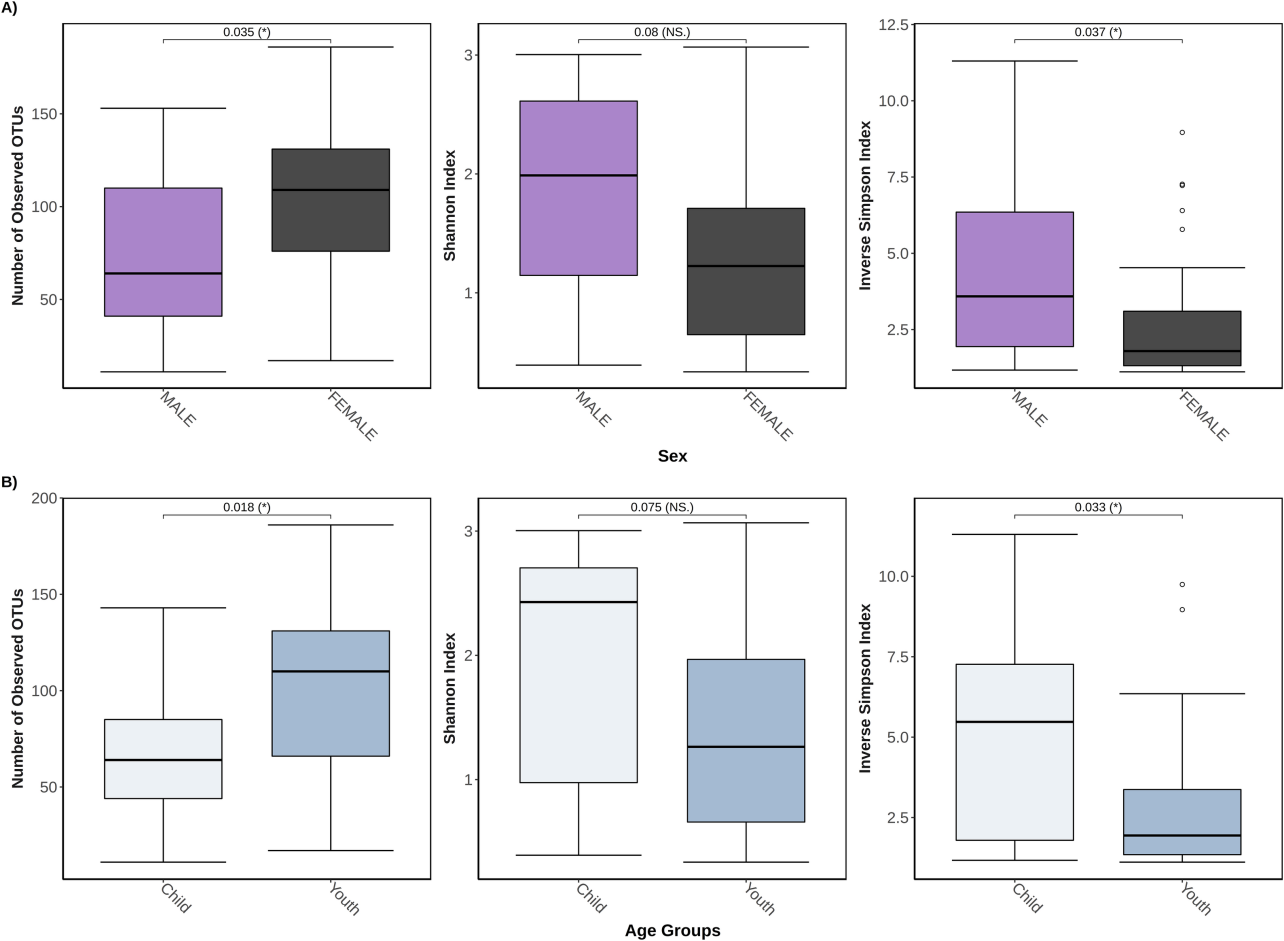
Three different α-diversity metrics were calculated for 46 study participants stratified by either sex or age groups. In **(A)** the total study cohort is stratified by sex (*n* = 29 female participants and *n* = 17 male participants) and is measured for the number of observed operational taxonomic units (OTUs) that represent unique species, also known as the richness, Shannon diversity index, which accounts for richness and abundances but weighs more toward richness, and the inverse Simpson diversity index which accounts for richness and abundances but weighs more toward abundance; **(B)** shows a stratification by age groups of child (≤14 years old, *n* = 13), and youth (between 15 and 24 years old, *n* = 33).

From the complementary analyses (when all participants were combined), some of the diversity metrics differed by age and sex (Figures 4A,B). Female participants exhibited a higher overall fungal richness but a lower inverse Simpson diversity index than male participants (both *p* ≤ 0.05 [adjusted]), with no significant differences observed for the Shannon diversity index (*p* = 0.08 [adjusted]). Similarly, for age, youth (age: 15+ years) exhibited a higher overall fungal richness but a lower inverse Simpson diversity index than children (age: ≤14 years) (both *p* ≤ 0.05 [adjusted]), with no significant differences observed for the Shannon diversity index (*p* = 0.08 [adjusted]). Significant individual heterogeneity was detected when testing the mycobiota between individuals within each group (POMS/monoADS/unaffected controls, sex and age groups, Kruskal–Wallis ANOVA, all *p* < 0.05).

**Figure 5:**
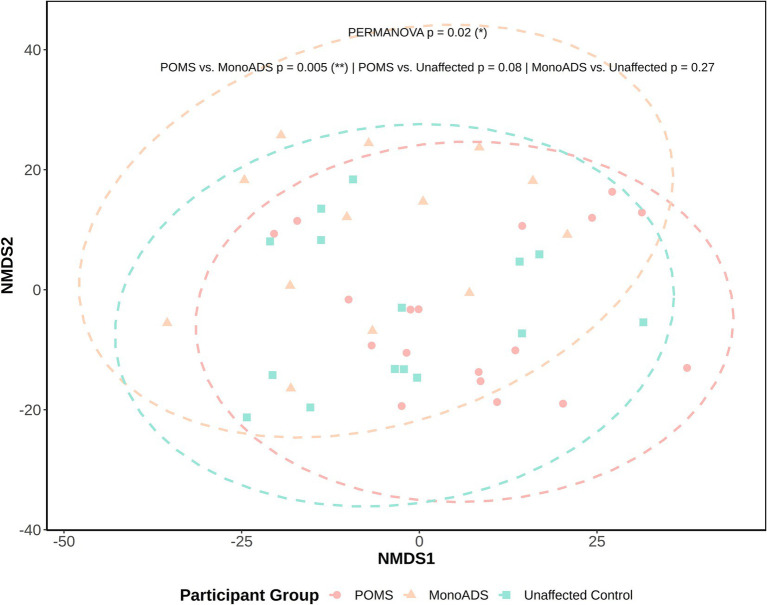
*β*-Diversity analysis by non-metric multidimensional scaling for the pediatric-onset multiple sclerosis cases, monophasic acquired demyelinating syndrome, and unaffected control participants. Calculations are based on central log ratio (CLR) transformed counts. Ellipses represent 95% confidence intervals, and the Euclidean distance is used for calculations.

For *β*-diversity, while no obvious or distinct clustering patterns could be visually observed between the MS, monoADS, and unaffected participants (Figure 5), quantification by PERMANOVA suggested a significant difference among the three groups (*p* = 0.02). Specifically, the pairwise comparisons indicated a significant difference between the monoADS and POMS (*p* = 0.005 [adjusted]), but not between the unaffected and POMS (*p* = 0.08 [adjusted]) or monoADS (*p* = 0.27 [adjusted]) participants.

Further examination of β-diversity upon stratifying diagnoses by sex or age group yielded no visible clustering between any of the stratified groups, although these observations should be interpreted with due caution as some group sizes were very small (Supplementary Figures S5, S6). Naturally, no formal statistical analyses were performed with these small groups (e.g., POMS male *n* = 3, and POMS child *n* = 1).

### 3.5 Differential abundance analysis

**Figure 6:**
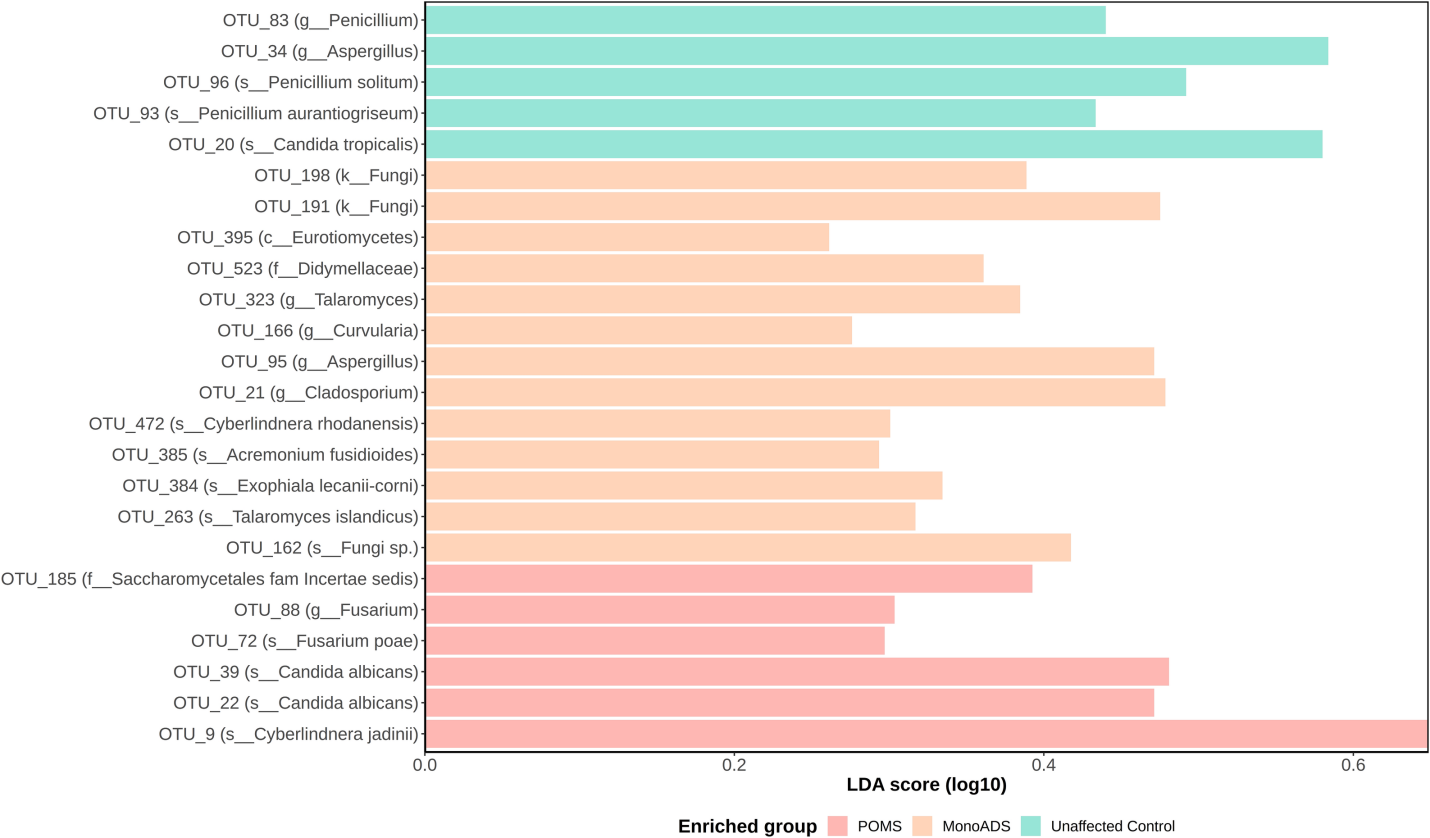
Operational taxonomic unit (OTU) differential abundance analysis by linear discriminant analysis effect size (LEfSe) for pediatric-onset multiple sclerosis cases, monophasic acquired demyelinating syndrome, and unaffected control participants. Significantly predicted OTUs are subject to linear discriminant analysis modeling, and effect size is determined to find the strongest class association. Effect sizes are converted into a score and plotted on a log10 scale.

Analysis of taxonomic abundances between the groups yielded no significant differences by the ALDEx2 analysis. However, the LEfSe analysis predicted potential marker organisms (Figure 6). The species *C. albicans*, *Cyberlindera jadinii*, and *Fusarium poae* were predicted as the markers specific to the POMS participants. *Acremonium fusidioides*, *Cyberlindnera rhodanensis*, *Exophiala lecanii-corni*, *Talaromyces islandicus*, and an unclassified fungal species were predicted to be markers of monoADS by LEfSe. In unaffected participants, *Penicillium aurantiogriseum* and *Penicillium solitum* were predicted. A BLAST analysis of species-level taxa identified by LEfSe yielded multiple unclassified identities noted in Supplementary Table S1.

Using the updated UNITE v10.0 fungal ribosomal database in an attempt to reclassify the large number of *Unknown* and *unclassified Eurotiales* genera particularly evident in the monoADS participants resulted in more distinct fungal identities (Supplementary Table S2). However, issues related to possible ambiguous identification remained. For example, OTU 483, previously labeled “*Unknown*,” was reclassified possibly as *Fungi gen Incertae sedis* or *Filobasidium*, OTU 169 as *Pleosporales gen Incertae sedis* or *Malassezia*, etc.

## 4 Discussion

Few studies have characterized the gut fungal profile in persons with MS and fewer in those with pediatric-onset MS (POMS). We also compared participants with POMS to both monoADS and unaffected control participants. While there was some indication of differences among the three groups for both the mycobiome *α*- and *β*-diversity metrics when each participant group was compared to the other, only *β*-diversity showed a significant difference, specifically between the monoADS and POMS participants [*p* = 0.005 (adjusted)]. Moreover, at the genus taxa level, 7 of the top 10 most abundant genera were similar between all three groups, with *Saccharomyces* and *Candida* being in the highest abundance. These are also commonly observed in other gut mycobiome studies and appear largely in transit, passing from food into the stool. In the POMS cases, the *Agaricus* genus dominated, primarily due to *A. bisporus* which is considered transient and commonly consumed, for example, as the white button mushroom (Webster and Weber, 2007; Auchtung et al., 2018). However, predictive modeling of differential abundance showed that *C. albicans, C. jadinii,* and *F. poae* were strongly associated with the POMS participants. Our study provides novel insight into the fungal gut mycobiota in POMS. While findings indicate that the gut mycobiota of participants with POMS may comprise fungi that are considered rather transient (Auchtung et al., 2018), differential predictive analyses were also suggestive of rare or under-detected fungal markers being of potential importance, warranting consideration in the future mycobiome-MS-related studies.

We found only two other studies—one study by Shah et al., 2021 and another by Yadav et al., 2022—with which we compared our findings. Both studies explored the gut mycobiome in adults with MS compared to unaffected controls, recruiting between 20 and 25 MS participants and between 22 and 33 unaffected controls. Despite the modest size of these studies and the types of participants included (adults vs. youth/children), some consistency in findings was observed across the studies. For example, in both of these previous studies, the gut mycobiota of the MS cases and controls alike were dominated by the presence of the phylum Ascomycota. Our study also observed the same overwhelming presence of Ascomycota which represented over 80% of the relative abundance of the gut mycobiome for our POMS, monoADS, and unaffected controls. Both previous studies also identified Basidiomycota as predominant among their MS cases and controls. While we also found Basidiomycota, our findings suggested that it was more abundant in the POMS cases compared to the other participants. Of the top 10 most abundant genera, we observed *Saccharomyces* at the highest abundance in all three groups, in addition to *Aspergillus, Candida*, *Cladosporium*, *Mucor*, and *Penicillium,* which were also seen in high abundance within both previous studies (Shah et al., 2021; Yadav et al., 2022). This consensus of fungal genera found between studies suggests there is a commonality composing the human gut mycobiota regardless of participant grouping. There is a potential that rare or under-detected fungi may better explain the pathogenesis of MS.

A majority of the top 10 represented genera overlapped among the three participant groups in our study, but they differed in the proportionality of specific genera. Interestingly, we detected a distinctly abundant genus within each of the groups: *Agaricus* in POMS (N_reads_ = 456,760 for POMS, 87 for monoADS, and 1,777 for unaffected participants), *Phoma* in monoADS (N_reads_ = 46 for POMS, 946 monoADS, and 102 for controls), and *Exophiala* in unaffected participants (N_reads_ = 1,240 for POMS, 150 monoADS, and 6,059 for controls). Further examination of the data showed that *A. bisporus is* composed of 99.9% of the *Agaricus* genus in POMS participants. *A. bisporus* is edible and commonly consumed as the white button mushroom (Webster and Weber, 2007). Although the *Agaricus* genus comprised only 0.0198% of the total genera in monoADS participants and 0.062% in unaffected controls, *A. bisporus* constituted a majority of the *Agaricus* spp. (100% for the monoADS and 89.8% for the unaffected controls). This echoes the current understanding that the gut mycobiome is likely largely impacted by diet and is transient in nature (Auchtung et al., 2018). Consistent with this, we also detected *Saccharomyces* spp. and *Candida* spp. at the highest proportion in all three groups. These are the two common fungal species reported as the transient residents of the gut acquired through diet (Auchtung et al., 2018).

Some similarities in terms of mycobiome diversity findings were observed across studies, as all three studies (including ours) reported at least some differences between participant groups. For example, *β*-diversity—the communal differences between groups differed between the adult MS and control participants in both previous studies (Shah et al., 2021; Yadav et al., 2022). While we also observed a difference between our three participant groups, this only remained significant when our POMS were compared to our monoADS individuals (neither were significant when compared to our unaffected participants). However, visually, *β*-diversity analysis revealed no distinctive clustering among our participant groups. We also investigated heterogeneity between individuals within the stratified groups (POMS/monoADS/unaffected controls, sex, and age groups), and significant differences were detected. Whether this occurred by chance alone was unclear, although others have observed that the gut mycobiota was highly variable within and between participants enrolled in the Human Microbiome Project (Nash et al., 2017). It is possible that with larger sample sizes, some of these diversity-related differences will become more apparent.

Our study also examined if there was a difference in *α*-diversity between male participants vs. female participants, and by age groups (child ≤14 years of age vs. youth age range 15–24 years). We did detect significant differences in the richness of both stratifications by sex and age groups. However, when we examined differences in fungal diversity, only the inverse Simpson diversity measure indicated differences between male participants and female participants, as well as between children and youth. Since both Shannon and inverse Simpson indices measure diversity in similar ways—they both account for the overall richness as well as the relative abundance of each species—the significance detected by the inverse Simpson index and not the Shannon index, can perhaps be attributed to their contrast in scale calculations, as the inverse Simpson is scaled toward higher abundances while the Shannon index favors richness (Roswell et al., 2021). Few have explored the relationship between age, sex, and the gut mycobiome. A study by Strati et al., 2016 recruited 111 “healthy” Italian volunteers and reported that the gut mycobiota exhibited higher richness in 48 children (3–10 years) and 24 adolescents (11–17 years) when compared to 21 adults (≥18). This explanation of higher richness at lower age groups during a period of eubiosis in healthy individuals—when intestinal homeostasis is maintained, and a balanced composition of microbes exists—may explain why our POMS cohort has a less rich—but not statistically significant—mycobiota compared to unaffected participants (Al-Rashidi, 2022). This is because the mycobiota in our POMS participants may be undergoing dysbiosis—the dysregulation of homeostasis associated with an imbalanced microbial composition—which could lower the overall number of unique fungi as compared to unaffected controls. Furthermore, when examining *β*-diversity by participant diagnosis paired with their sex or age group, there was no visual indication that a common mycobiota existed between groups (e.g., POMS male and unaffected control male). Our findings suggest that age and sex should be taken into consideration when conducting future mycobiota analyses, as we found female individuals and youth have higher fungal richness but lower diversity when compared to male individuals and children. Future studies involving a larger cohort of individuals are required to investigate the associations between sex or age and the gut mycobiota across different disease groups.

We further explored the idea that rare species play an important role by employing LEfSe predictive analyses. We focus only on significant OTUs assignable to a known fungal species using the UNITE database because of multiple ambiguous taxonomic classifications when attempting to resolve identities using the RefSeq ITS database. Our LEfSe analysis predicted *F*. *poae* as a possible marker of relevance to our POMS participants. This organism has a role in agriculture as a plant pathogen that commonly contaminates animal feed (Yang et al., 2014; Ghimire et al., 2021; Tan et al., 2021). Additionally, the genus *Fusarium* has the potential to produce mycotoxins, which have been shown *in vitro* to inhibit sphingosine biosynthesis important for the formation of sphingomyelin lining the neuronal axon (Norred et al., 1992). Our LEfSe analysis also predicted *C. jadinii* to be associated with our POMS cohort, and this organism can be identified under the telomorphic name of *Candida utilis*. Several *Candida* species are known opportunistic pathogens able to cause invasive Candidiasis (Goering et al., 2019).

Perhaps the majority of interesting LEfSe predictive association with POMS participants was *C. albicans*. Although *C. albicans* is widely recognized to be part of the human gut mycobiome, it is also a known opportunistic human pathogen (Nash et al., 2017; Auchtung et al., 2018; Goering et al., 2019). Furthermore, *C. albicans* has previously been detected in the cerebrospinal fluid and in postmortem brain tissue samples from people with MS (Ramos et al., 2008; Pisa et al., 2011, 2013; Alonso et al., 2018). Additionally, in the mouse model of MS, experimental autoimmune encephalomyelitis, it has been shown that *C. albicans* exacerbates inflammation and demyelination, but a decrease of *C. albicans* alleviated these effects (Fraga-Silva et al., 2015). In studies of other neurological diseases, *C. albicans* was also found to play a role in the mice model of Alzheimer’s disease (Wu et al., 2019). Although evidence remains rather limited, it would be of value for future studies to evaluate the role of *C. albicans* in MS, especially because spp. from the *Candida* genus are known to coexist as a large part of our normal mycobiota without disease, until perhaps there is overt dysregulation in the hosts’ immune system (Nash et al., 2017; Goering et al., 2019).

In our monoADS cohort, LEfSe analysis identified *A. fusidioides*, *E. lecanii-corni*, and *T. islandicus* as predicted species. Relatively little information can be found on *A. fusidioides* but this fungus has been shown to produce compounds able to inhibit the growth of HL-60 human leukemia cells *in vitro* (An et al., 2016). In a rare isolated case of phaeohyphomycosis, which has a documented mortality rate of ~70% in CNS infections, *E. lecanii-corni* was identified as the causative agent (Revankar et al., 2004; Lee et al., 2016). Furthermore, studies show that *T. islandicus* is capable of producing the mycotoxin, cyclic pentapeptide cyclochlorotine, which is said to have mutagenic and toxic effects on humans (Schafhauser et al., 2015). Although these organisms were predicted as significant markers, more research is necessary to understand their role in monoADS.

The *Penicillium* genus was largely predicted to associate with unaffected control participants according to our LEfSe analysis, namely, *P. aurantiogriseum*, *P. solitum*, *P. aurantiogriseum*. Of interest, *P. solitum* has been reported to produce many types of metabolites important for sustaining human health, such as compactin, benzodiazepine alkaloids, and meroterpenoids (Boruta et al., 2018).

Methods to study the fungal mycobiome require further development to better understand the role of the gut mycobiota in MS. Our study provides a high-level characterization of the fungal gut community for participants with POMS, compared to monoADS and unaffected controls. POMS is a rare condition that limited the size of our study, as did the necessary step of removing any study participant in the post-data filtration stage to prevent bias by the low-quality reads (Nilsson et al., 2019a; Cao et al., 2021). We were also unable to substratify our cohorts to explore the possible impact of, for example, the use of disease-modifying drugs. In the future, larger studies are also needed to explore the effects of sex and age on the gut mycobiome composition. In addition to a larger sample size, future studies could be enhanced by exploring the relationship between the fungal and bacterial gut microbiota in POMS.

The current cross-sectional study would benefit from the longitudinal collection of participant stool to, for example, investigate the potential role of age on the gut mycobiota composition over time. As expected in a cohort of individuals with POMS early in their disease course, disability scores, indicated by the Expanded Disability Status Scale, were low (scores averaged ~1–1.5). Longitudinal sample collection would also allow monitoring of disease activity or disability progression and the relationship with the gut mycobiota composition, including investigation of differences when stratifying by sex. Furthermore, while we purposely chose the commonly used internal transcribed spacer 2 (ITS2) region (also employed by one of the two previous MS-related mycobiome studies), it is worth noting that the choice of primer will influence the amplification of different fungal phyla and will affect comparisons between studies (Frau et al., 2019; Mbareche et al., 2021; Yadav et al., 2022).

We also acknowledge that comprehensive and well-curated reference databases are critical for mycobiome studies. A proportion of fungal sequences in our study were unclassified fungal organisms in each of the three groups (POMS, monoADS, and unaffected controls), exposing a limitation of the currently available database for fungal taxonomic assignment (Webster and Weber, 2007). Our study was able to identify certain fungi at the species level using the RefSeq ITS database that could not be definitively assigned using the UNITE database. Although the use of the RefSeq ITS database did provide a higher taxonomic resolution for the majority of the significant OTUs from our LEfSe analysis, some OTUs could not be unambiguously classified to a single species level. In addition, our attempt to reclassify a large proportion of the *Unknown* and *unclassified* genera, which were particularly evident in the monoADS participants, resulted in improved resolution as we conducted a local BLAST against the latest version of the UNITE fungal ribosomal database. However, it should be noted that the fungal nomenclature system is experiencing an ongoing period of reorganization with many fungal organisms yet to be discovered and characterized (Siqueira et al., 2018; Goering et al., 2019; Nilsson et al., 2019b).

## 5 Conclusion

Although modest in size, our study provides novel insight into the fungal gut mycobiota in persons with and without POMS. While our findings suggested that the gut mycobiome of POMS participants may be largely dominated by transient colonizers, the possibility that rare or under-detected fungal markers may play a role in the pathophysiology of POMS warrants further consideration. Our findings guide future “-omics” based investigations in MS, which ideally include a more granular assessment of the gut mycobiome, especially in pediatric-onset participants, its functional potential, and interaction(s) with the wider gut microbiome community.

## Data Availability

The data presented in the study are deposited in the NCBI repository (https://www.ncbi.nlm.nih.gov/), accession number PRJNA1005564.
