# Supplementary material for: The fungal gut microbiota in pediatric-onset multiple sclerosis
Source: Front Microbiol. 2024 Dec 18;15:1258978. doi: 10.3389/fmicb.2024.1258978 (PMC11688249; doi:10.3389/fmicb.2024.1258978)
Supplement: Supplementary file 1 [file Supplementary_file_1.docx]

Supplementary Material

# Supplementary Figures and Tables

## Supplementary Figures
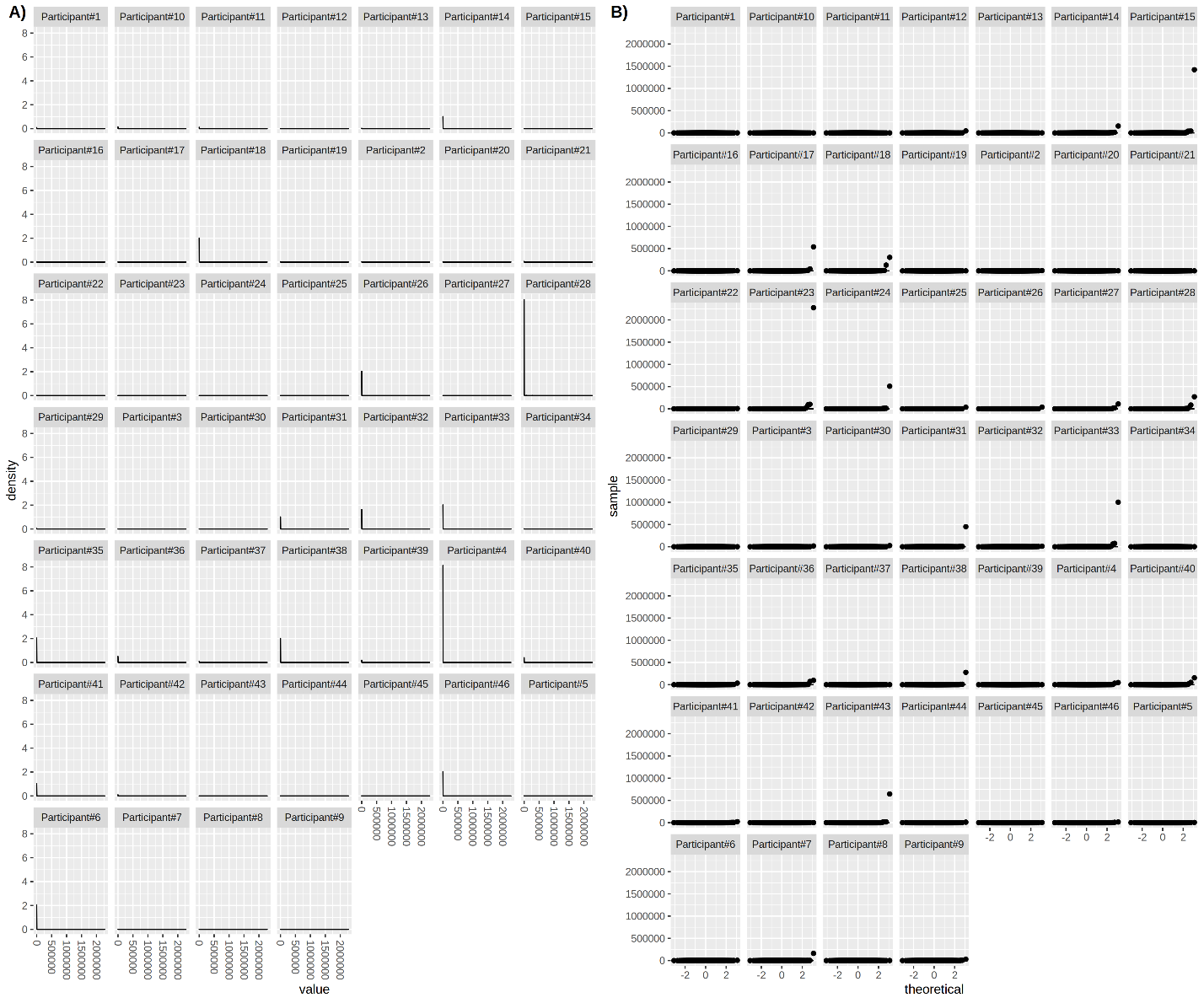


**Supplementary Figure 1.** Density distributions and quantile-quantile plots of untransformed relative abundances. The sequencing reads from the 46 participants (pediatric-onset multiple sclerosis cases, monophasic acquired demyelinating syndrome, and unaffected controls) were examined for normality. In the left figure **A)** 46 density distributions correspond to each study individual; **B)** 46 quantile-quantile plots correspond to each study individual.


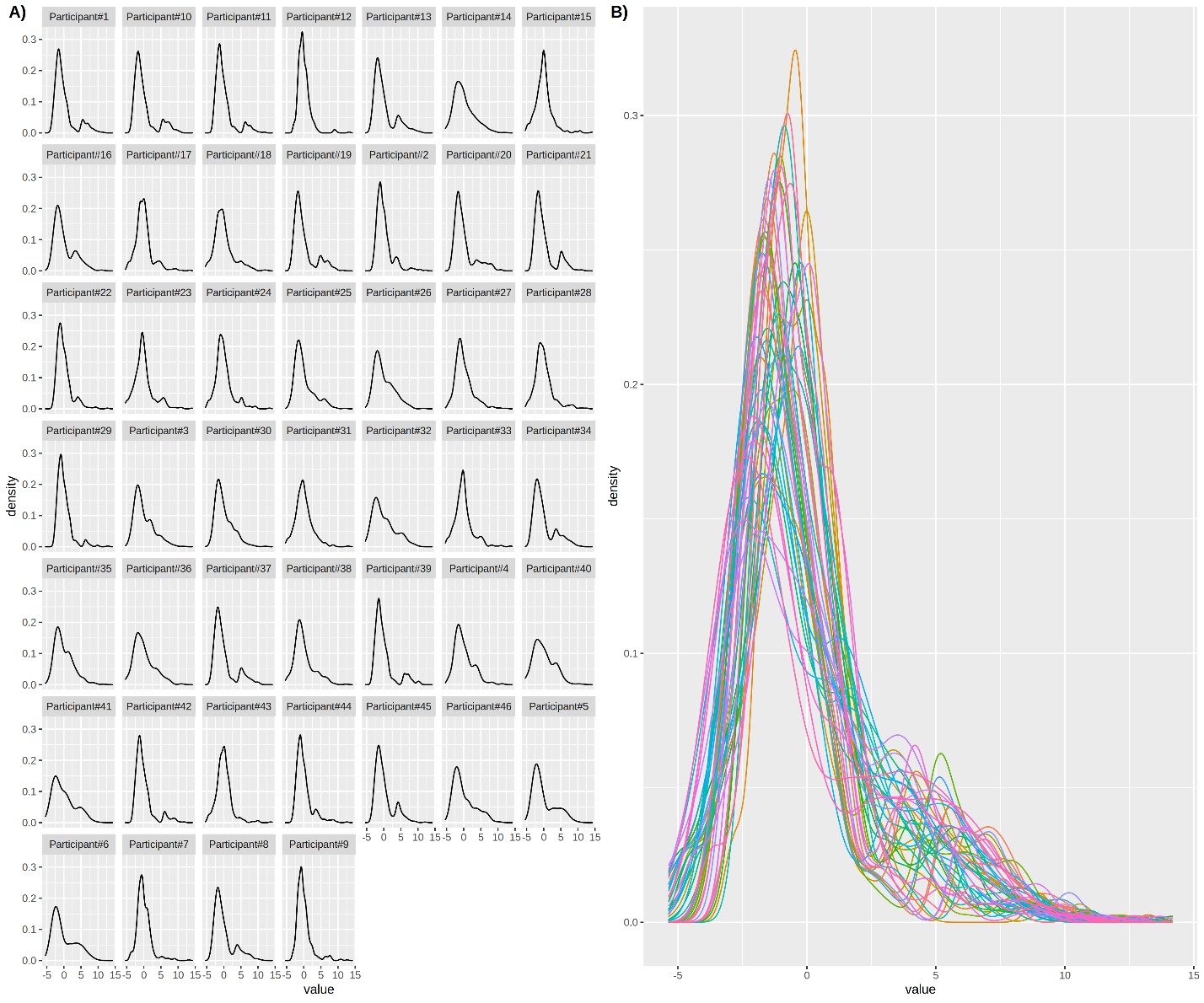


**Supplementary Figure 2.** Density distributions of CLR transformed relative abundances. The sequencing reads from the 46 participants (pediatric-onset multiple sclerosis cases, monophasic acquired demyelinating syndrome, and unaffected controls) were transformed using the CLR method and examined for normality. In the left figure **A)** 46 density distributions corresponding to each study individual; **B)** All 46 density distributions from **A** aggregated into a single plot.


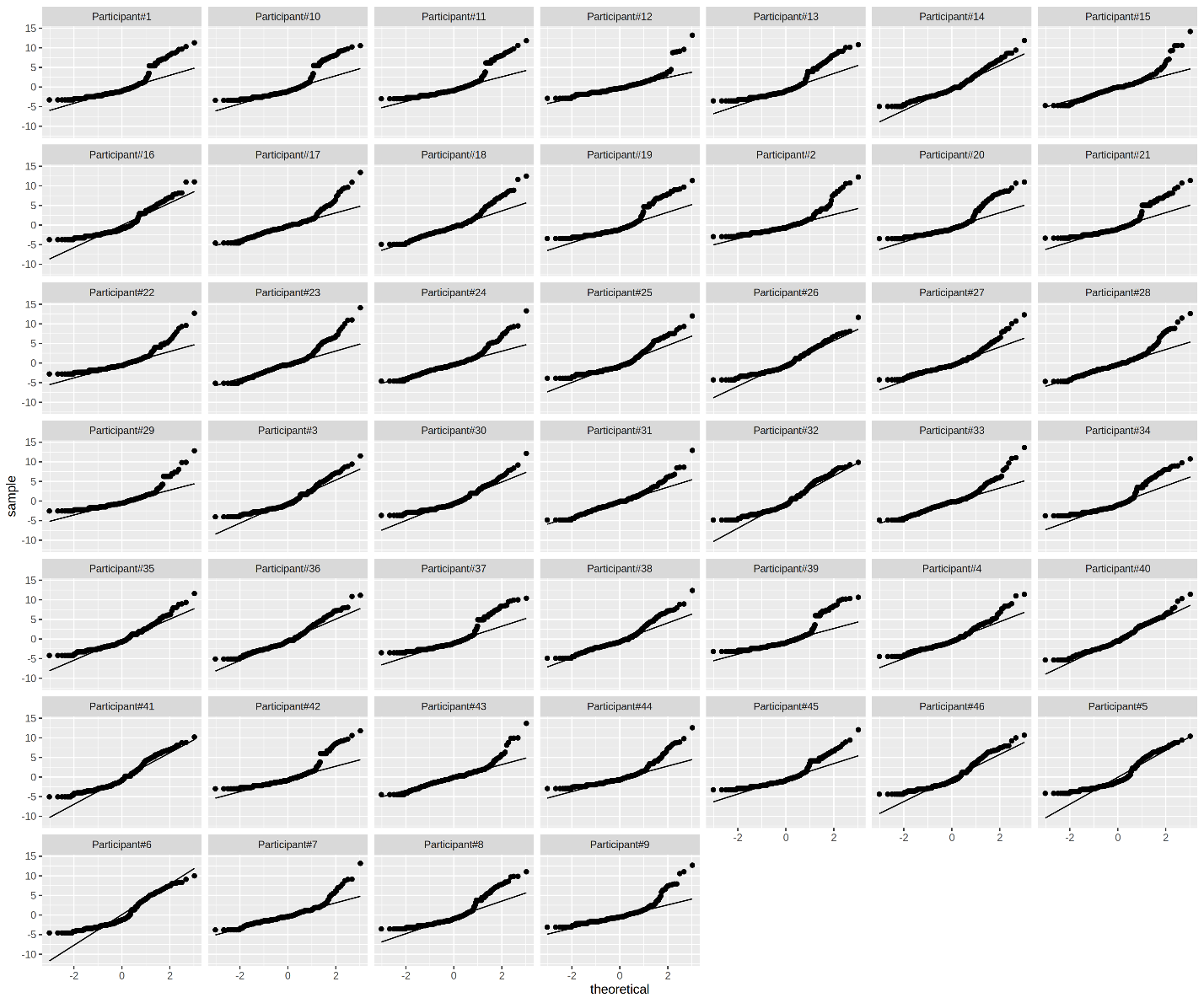


**Supplementary Figure 3.** Quantile-quantile plots of CLR transformed relative abundances. The sequencing reads from a 46-member cohort composed of pediatric-onset multiple sclerosis cases, monophasic acquired demyelinating syndrome, and unaffected control participants were transformed using the CLR method and examined for normality.


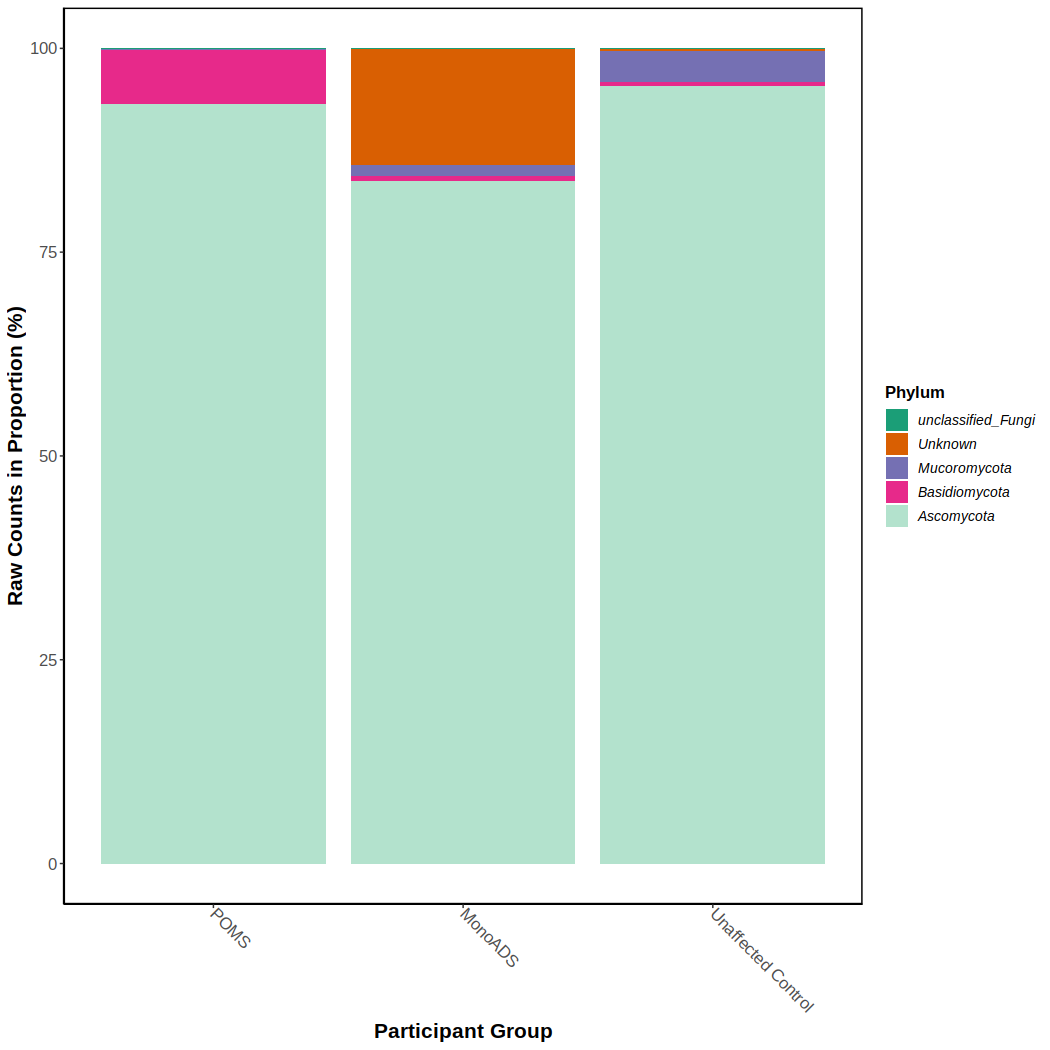


**Supplementary Figure 4.** Stacked bar plot showing the proportion of fungal phyla found for the 18 pediatric-onset multiple sclerosis (MS) cases, 13 monophasic acquired demyelinating syndrome (monoADS), and 15 unaffected control participants.

**
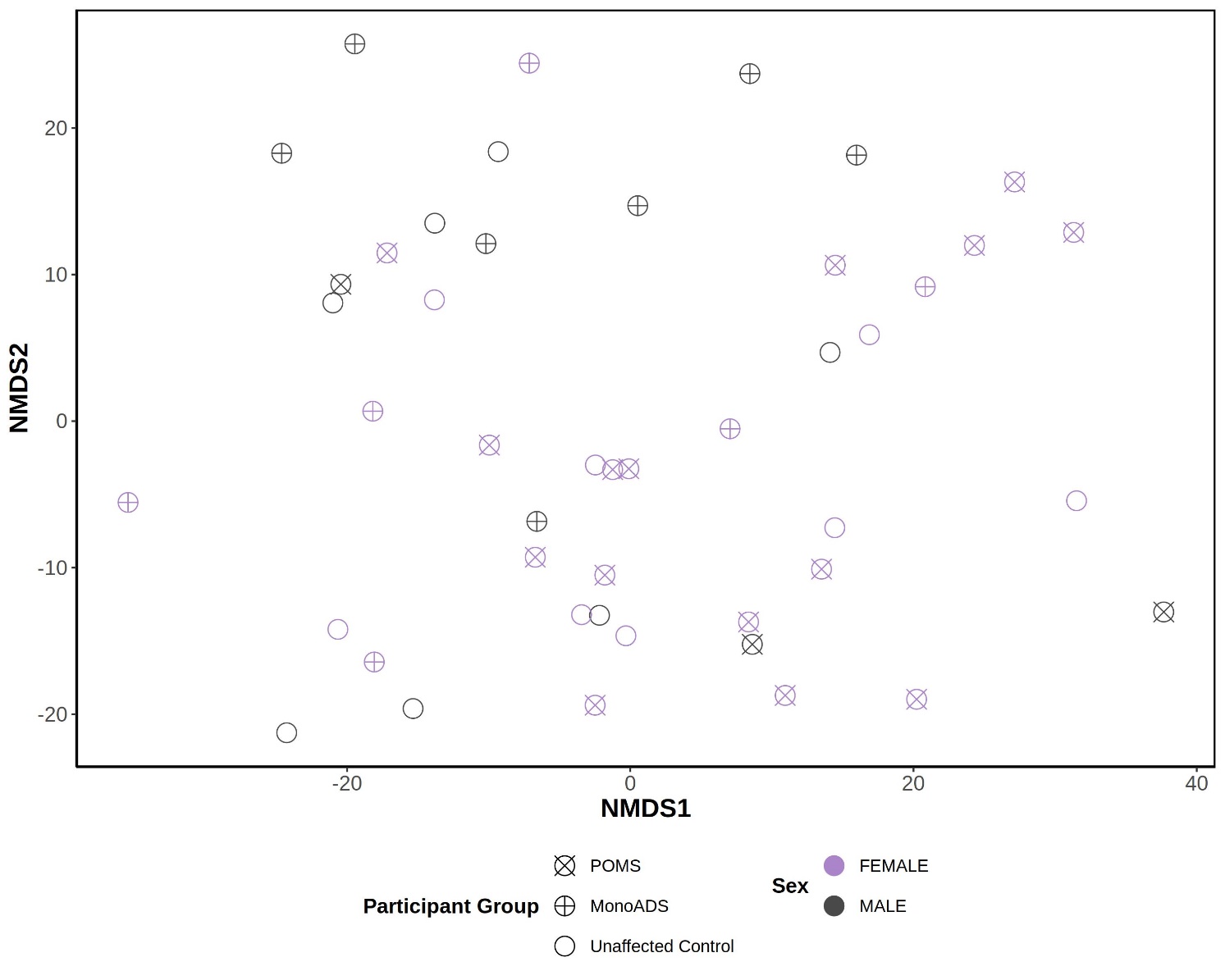
Supplementary Figure 5.** Beta diversity analysis by non-metric multidimensional scaling for the pediatric-onset multiple sclerosis cases, monophasic acquired demyelinating syndrome, and unaffected control participants paired with participant sex.


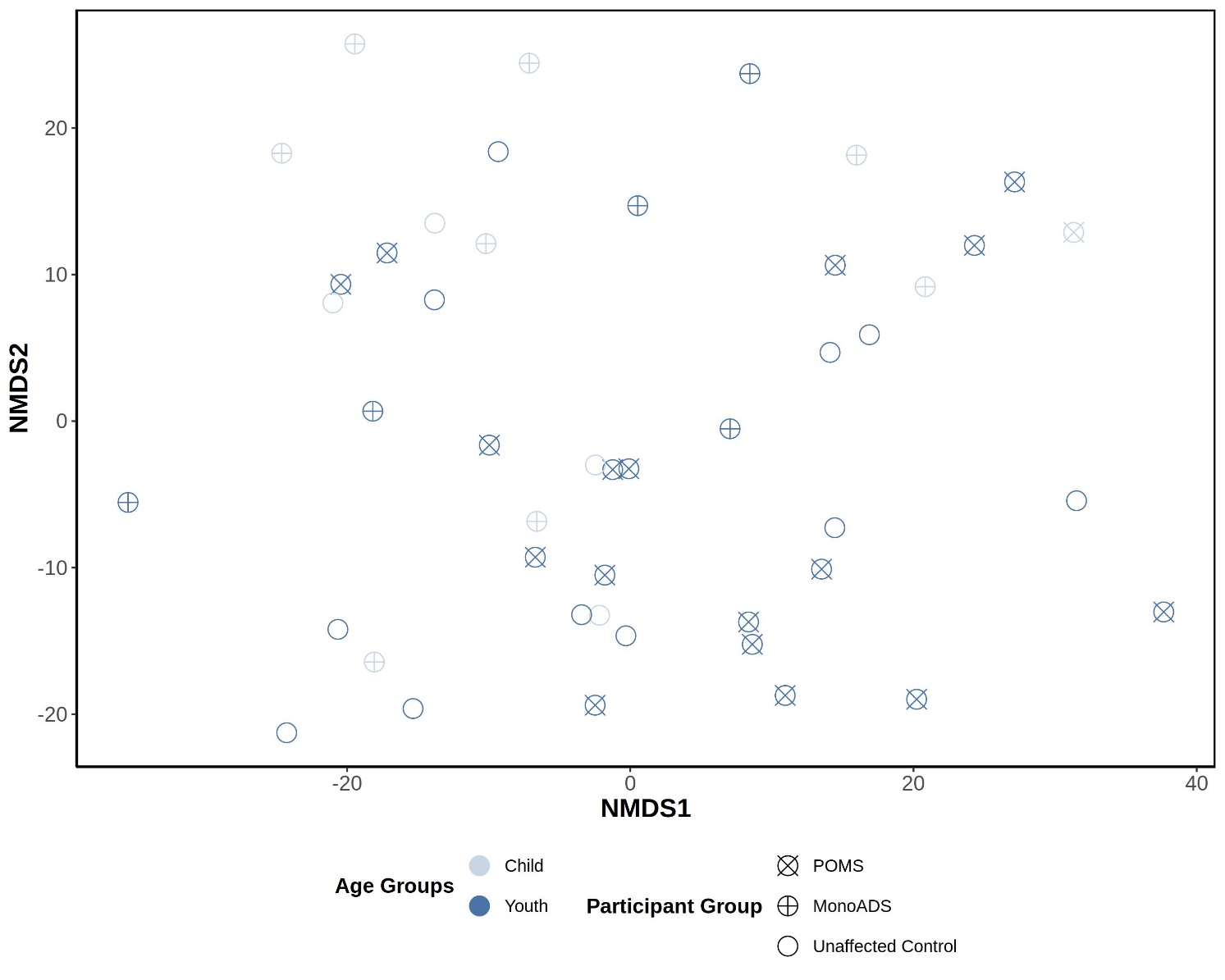


**Supplementary Figure 6.** Beta diversity analysis by non-metric multidimensional scaling for the pediatric-onset multiple sclerosis cases, monophasic acquired demyelinating syndrome, and unaffected control participants paired with participant age group.

## Supplementary Tables

**Supplementary Table 1.** BLAST identities of organisms predicted above a species taxonomic level from LEfSe against the RefSeq ITS database

| **OTU** | **UNITE Taxon ID** | **BLAST Taxon ID** | **BLAST Accession** | **%Identity** | **QCovHSP^a^** | **Expected Value** |
| --- | --- | --- | --- | --- | --- | --- |
| OTU 166 | g__Curvularia | *Curvularia alcornii MFLUCC 10-0703* | NR_137091 | 100 | 100 | 1.67E-81 |
| OTU 185 | f__Saccharomycetales fam Incertae sedis | *Candida africana* | NR_138276 | 91.333 | 100 | 9.60E-54 |
| OTU 185 | f__Saccharomycetales fam Incertae sedis | *Candida albicans* | NR_125332 | 91.333 | 100 | 9.60E-54 |
| OTU 191 | k__Fungi | *Cladosporium angustiherbarum* | NR_152286 | 97.297 | 100 | 1.20E-67 |
| OTU 191 | k__Fungi | *Cladosporium aggregatocicatricatum* | NR_152300 | 97.297 | 100 | 1.20E-67 |
| OTU 191 | k__Fungi | *Cladosporium rhusicola* | NR_152299 | 97.297 | 100 | 1.20E-67 |
| OTU 191 | k__Fungi | *Cladosporium puyae* | NR_152298 | 97.297 | 100 | 1.20E-67 |
| OTU 191 | k__Fungi | *Cladosporium versiforme* | NR_152297 | 97.297 | 100 | 1.20E-67 |
| OTU 191 | k__Fungi | *Cladosporium subcinereum UTHSC DI-13-257* | NR_148193 | 97.297 | 100 | 1.20E-67 |
| OTU 191 | k__Fungi | *Cladosporium antarcticum* | NR_121332 | 97.297 | 100 | 1.20E-67 |
| OTU 191 | k__Fungi | *Cladosporium phlei* | NR_120013 | 97.297 | 100 | 1.20E-67 |
| OTU 191 | k__Fungi | *Cladosporium cucumerinum MUCL 10092* | NR_119841 | 97.297 | 100 | 1.20E-67 |
| OTU 191 | k__Fungi | *Cladosporium chubutense* | NR_119728 | 97.297 | 100 | 1.20E-67 |
| OTU 191 | k__Fungi | *Cladosporium variabile* | NR_119663 | 97.297 | 100 | 1.20E-67 |
| OTU 191 | k__Fungi | *Cladosporium tenellum* | NR_119662 | 97.297 | 100 | 1.20E-67 |
| OTU 191 | k__Fungi | *Cladosporium spinulosum* | NR_119660 | 97.297 | 100 | 1.20E-67 |
| OTU 191 | k__Fungi | *Cladosporium macrocarpum* | NR_119657 | 97.297 | 100 | 1.20E-67 |
| OTU 191 | k__Fungi | *Cladosporium herbarum* | NR_119656 | 97.297 | 100 | 1.20E-67 |
| OTU 191 | k__Fungi | *Cladosporium allicinum* | NR_152266 | 97.297 | 100 | 1.20E-67 |
| OTU 191 | k__Fungi | *Cladosporium herbaroides* | NR_119655 | 97.297 | 100 | 1.20E-67 |
| OTU 191 | k__Fungi | *Cladosporium iridis* | NR_111271 | 97.297 | 100 | 1.20E-67 |
| OTU 198 | k__Fungi | *Clavispora lusitaniae* | NR_130677 | 100 | 100 | 5.26E-33 |
| OTU 21 | g__Cladosporium | *Cladosporium chasmanthicola CPC 21300* | NR_152307 | 100 | 100 | 2.56E-74 |
| OTU 21 | g__Cladosporium | *Cladosporium pseudochalastosporoides* | NR_152296 | 100 | 100 | 2.56E-74 |
| OTU 21 | g__Cladosporium | *Cladosporium welwitschiicola CPC 18648* | NR_152308 | 100 | 100 | 2.56E-74 |
| OTU 21 | g__Cladosporium | *Cladosporium parapenidielloides* | NR_152293 | 100 | 100 | 2.56E-74 |
| OTU 21 | g__Cladosporium | *Cladosporium longicatenatum* | NR_152291 | 100 | 100 | 2.56E-74 |
| OTU 21 | g__Cladosporium | *Cladosporium ipereniae* | NR_152290 | 100 | 100 | 2.56E-74 |
| OTU 21 | g__Cladosporium | *Cladosporium austroafricanum* | NR_152288 | 100 | 100 | 2.56E-74 |
| OTU 21 | g__Cladosporium | *Cladosporium angustiterminale* | NR_152287 | 100 | 100 | 2.56E-74 |
| OTU 21 | g__Cladosporium | *Cladosporium crousii UTHSC DI-13-247* | NR_148192 | 100 | 100 | 2.56E-74 |
| OTU 21 | g__Cladosporium | *Cladosporium phaenocomae* | NR_119950 | 100 | 100 | 2.56E-74 |
| OTU 21 | g__Cladosporium | *Cladosporium varians CPC 13658* | NR_119856 | 100 | 100 | 2.56E-74 |
| OTU 21 | g__Cladosporium | *Cladosporium subuliforme CPC 13735* | NR_119854 | 100 | 100 | 2.56E-74 |
| OTU 21 | g__Cladosporium | *Cladosporium exile CPC 11828* | NR_111532 | 100 | 100 | 2.56E-74 |
| OTU 21 | g__Cladosporium | *Cladosporium globisporum* | NR_111534 | 100 | 100 | 2.56E-74 |
| OTU 21 | g__Cladosporium | *Cladosporium myrtacearum CPC 14567* | NR_119849 | 100 | 100 | 2.56E-74 |
| OTU 21 | g__Cladosporium | *Cladosporium angustisporum CPC 12437* | NR_111530 | 100 | 100 | 2.56E-74 |
| OTU 21 | g__Cladosporium | *Cladosporium iranicum CPC 11554* | NR_111536 | 100 | 100 | 2.56E-74 |
| OTU 21 | g__Cladosporium | *Cladosporium funiculosum* | NR_119845 | 100 | 100 | 2.56E-74 |
| OTU 21 | g__Cladosporium | *Cladosporium flabelliforme CPC 14523* | NR_119844 | 100 | 100 | 2.56E-74 |
| OTU 21 | g__Cladosporium | *Cladosporium exasperatum CPC 14638* | NR_119843 | 100 | 100 | 2.56E-74 |
| OTU 21 | g__Cladosporium | *Cladosporium montecillanum* | NR_152292 | 100 | 100 | 2.56E-74 |
| OTU 21 | g__Cladosporium | *Cladosporium gamsianum CPC 11807* | NR_111533 | 100 | 100 | 2.56E-74 |
| OTU 21 | g__Cladosporium | *Cladosporium australiense CPC 13226* | NR_119837 | 100 | 100 | 2.56E-74 |
| OTU 21 | g__Cladosporium | *Cladosporium asperulatum CPC 14040* | NR_119836 | 100 | 100 | 2.56E-74 |
| OTU 21 | g__Cladosporium | *Cladosporium pini-ponderosae* | NR_119730 | 100 | 100 | 2.56E-74 |
| OTU 21 | g__Cladosporium | *Cladosporium colombiae* | NR_119729 | 100 | 100 | 2.56E-74 |
| OTU 21 | g__Cladosporium | *Cladosporium silenes* | NR_111270 | 100 | 100 | 2.56E-74 |
| OTU 323 | g__Talaromyces | *Talaromyces wortmannii* | NR_172039 | 99.419 | 100 | 6.44E-86 |
| OTU 34 | g__Aspergillus | *Aspergillus tubingensis NRRL 4875* | NR_131293 | 100 | 100 | 2.88E-84 |
| OTU 34 | g__Aspergillus | *Aspergillus costaricensis* | NR_103604 | 100 | 100 | 2.88E-84 |
| OTU 34 | g__Aspergillus | *Aspergillus luchuensis KACC 46772* | NR_135449 | 100 | 100 | 2.88E-84 |
| OTU 395 | c__Eurotiomycetes | *NA* | NA | NA | NA | NA |
| OTU 523 | f__Didymellaceae | *Dimorphoma saxea* | NR_157421 | 97.368 | 100 | 7.38E-70 |
| OTU 83 | g__Penicillium | *Penicillium neoechinulatum* | NR_163549 | 100 | 100 | 1.31E-82 |
| OTU 83 | g__Penicillium | *Penicillium hirsutum* | NR_163544 | 100 | 100 | 1.31E-82 |
| OTU 83 | g__Penicillium | *Penicillium robsamsonii* | NR_144866 | 100 | 100 | 1.31E-82 |
| OTU 83 | g__Penicillium | *Penicillium thymicola* | NR_137883 | 100 | 100 | 1.31E-82 |
| OTU 83 | g__Penicillium | *Penicillium marinum* | NR_137882 | 100 | 100 | 1.31E-82 |
| OTU 83 | g__Penicillium | *Penicillium compactum CGMCC 3.15411* | NR_144844 | 100 | 100 | 1.31E-82 |
| OTU 83 | g__Penicillium | *Penicillium concentricum* | NR_138334 | 100 | 100 | 1.31E-82 |
| OTU 83 | g__Penicillium | *Penicillium viridicatum FRR 963* | NR_119496 | 100 | 100 | 1.31E-82 |
| OTU 83 | g__Penicillium | *Penicillium verrucosum FRR 965* | NR_119495 | 100 | 100 | 1.31E-82 |
| OTU 83 | g__Penicillium | *Penicillium expansum ATCC 7861* | NR_077154 | 100 | 100 | 1.31E-82 |
| OTU 83 | g__Penicillium | *Penicillium italicum* | NR_163528 | 100 | 100 | 1.31E-82 |
| OTU 83 | g__Penicillium | *Penicillium tricolor* | NR_077206 | 100 | 100 | 1.31E-82 |
| OTU 83 | g__Penicillium | *Penicillium polonicum* | NR_103687 | 100 | 100 | 1.31E-82 |
| OTU 83 | g__Penicillium | *Penicillium albocoremium IBT 10682* | NR_138271 | 100 | 100 | 1.31E-82 |
| OTU 88 | g__Fusarium | *Fusarium robustum* | NR_159851 | 97.987 | 100 | 7.21E-70 |
| OTU 95 | g__Aspergillus | *Aspergillus heterocaryoticus* | NR_163674 | 100 | 100 | 3.67E-83 |
| OTU 95 | g__Aspergillus | *Aspergillus costiformis* | NR_135434 | 100 | 100 | 3.67E-83 |
| OTU 95 | g__Aspergillus | *Aspergillus chevalieri NRRL 78* | NR_135340 | 100 | 100 | 3.67E-83 |

**a =** QcovHSP stands for query coverage per high scoring pair

**Supplementary Table 2.** Genus taxonomic level BLAST comparison of *Unknown* and *unclassified* organisms amongst the monoADS participants from the UNITE v8.2 database assigned in this study versus the latest v10.0 database

| **OTU** | **Original Taxon ID^a^** | **Updated Taxon ID^b^** | **Accession** | **%Identity** | **QCovHSP^c^** | **Expected Value** |
| --- | --- | --- | --- | --- | --- | --- |
| OTU 8 | *Unknown* | *g__Fusarium* | AB587010 | 100 | 100 | 3.89E-75 |
| OTU 8 | *Unknown* | *g__Fusarium* | GU721385 | 100 | 100 | 3.89E-75 |
| OTU 8 | *Unknown* | *g__Fusarium* | KY426419 | 100 | 100 | 3.89E-75 |
| OTU 11 | *Unknown* | *g__Geotrichum* | KF713521 | 100 | 100 | 3.63E-26 |
| OTU 11 | *Unknown* | *g__Geotrichum* | JX847744 | 100 | 100 | 3.63E-26 |
| OTU 11 | *Unknown* | *g__Geotrichum* | JX847770 | 100 | 100 | 3.63E-26 |
| OTU 16 | *Unknown* | *g__Fusarium* | AB586992 | 100 | 100 | 1.39E-74 |
| OTU 16 | *Unknown* | *g__Fusarium* | OR735591 | 100 | 100 | 1.39E-74 |
| OTU 16 | *Unknown* | *g__Fusarium* | EU754935 | 99.333 | 100 | 6.45E-73 |
| OTU 23 | *Unknown* | *g__Clavispora* | AY321464 | 100 | 100 | 3.55E-32 |
| OTU 23 | *Unknown* | *g__Clavispora* | FJ183442 | 100 | 100 | 3.55E-32 |
| OTU 23 | *Unknown* | *g__Clavispora* | KP764997 | 100 | 100 | 3.55E-32 |
| OTU 25 | *Unknown* | *g__Candida* | AJ549822 | 100 | 100 | 1.17E-48 |
| OTU 25 | *Unknown* | *g__Saccharomycetales_gen_Incertae_sedis* | AM160629 | 99.029 | 100 | 5.45E-47 |
| OTU 25 | *Unknown* | *g__Saccharomycetales_gen_Incertae_sedis* | HM589230 | 98.058 | 100 | 2.53E-45 |
| OTU 30 | *Unknown* | *g__Alternaria* | ON711922 | 100 | 94 | 5.30E-74 |
| OTU 30 | *Unknown* | *g__Alternaria* | KC146356 | 99.367 | 100 | 8.81E-77 |
| OTU 30 | *Unknown* | *g__Alternaria* | OL753661 | 98.101 | 100 | 6.86E-73 |
| OTU 33 | *Unknown* | *g__Penicillium* | AF033422 | 100 | 100 | 1.99E-83 |
| OTU 42 | *unclassified_Eurotiales* | *g__Penicillium* | UDB035155 | 100 | 100 | 7.11E-83 |
| OTU 42 | *unclassified_Eurotiales* | *g__Penicillium* | OR603045 | 100 | 100 | 7.11E-83 |
| OTU 48 | *Unknown* | *g__Calophoma* | KY742049 | 100 | 100 | 8.55E-77 |
| OTU 48 | *Unknown* | *g__Didymella* | MN973533 | 99.351 | 100 | 3.98E-75 |
| OTU 48 | *Unknown* | *g__Didymella* | MK836111 | 99.351 | 100 | 3.98E-75 |
| OTU 60 | *Unknown* | *g__Penicillium* | AY373933 | 98.701 | 97 | 1.91E-73 |
| OTU 60 | *Unknown* | *g__Penicillium* | UDB04225642 | 97.403 | 97 | 4.13E-70 |
| OTU 65 | *Unknown* | *g__Geotrichum* | KF713521 | 100 | 100 | 3.63E-26 |
| OTU 65 | *Unknown* | *g__Geotrichum* | JX847744 | 100 | 100 | 3.63E-26 |
| OTU 65 | *Unknown* | *g__Geotrichum* | JX847770 | 100 | 100 | 3.63E-26 |
| OTU 66 | *Unknown* | *g__Aspergillus* | MN339603 | 99.39 | 100 | 1.18E-80 |
| OTU 66 | *Unknown* | *g__Aspergillus* | KJ809565 | 98.788 | 100 | 5.50E-79 |
| OTU 66 | *Unknown* | *g__Penicillium* | KT758158 | 98.788 | 100 | 5.50E-79 |
| OTU 76 | *Unknown* | *g__Geotrichum* | KF713521 | 100 | 100 | 3.63E-26 |
| OTU 76 | *Unknown* | *g__Geotrichum* | JX847744 | 100 | 100 | 3.63E-26 |
| OTU 76 | *Unknown* | *g__Geotrichum* | JX847770 | 100 | 100 | 3.63E-26 |
| OTU 106 | *Unknown* | *g__Bezerromyces* | KX470392 | 100 | 100 | 1.88E-78 |
| OTU 113 | *Unknown* | *g__Clavispora* | AY321464 | 100 | 100 | 3.55E-32 |
| OTU 113 | *Unknown* | *g__Clavispora* | FJ183442 | 100 | 100 | 3.55E-32 |
| OTU 113 | *Unknown* | *g__Clavispora* | KP764997 | 100 | 100 | 3.55E-32 |
| OTU 129 | *Unknown* | *g__Symmetrospora* | AF444577 | 98.529 | 100 | 1.89E-99 |
| OTU 129 | *Unknown* | *g__Symmetrospora* | AF444546 | 98.529 | 100 | 1.89E-99 |
| OTU 129 | *Unknown* | *g__Symmetrospora* | MK050388 | 97.059 | 100 | 1.91E-94 |
| OTU 143 | *Unknown* | *g__Fusarium* | AB587010 | 100 | 100 | 3.89E-75 |
| OTU 143 | *Unknown* | *g__Fusarium* | GU721385 | 100 | 100 | 3.89E-75 |
| OTU 143 | *Unknown* | *g__Fusarium* | KY426419 | 100 | 100 | 3.89E-75 |
| OTU 157 | *Unknown* | *g__Clavispora* | AY321464 | 100 | 100 | 3.55E-32 |
| OTU 157 | *Unknown* | *g__Clavispora* | FJ183442 | 100 | 100 | 3.55E-32 |
| OTU 157 | *Unknown* | *g__Clavispora* | KP764997 | 100 | 100 | 3.55E-32 |
| OTU 169 | *Unknown* | *g__Pleosporales_gen_Incertae_sedis* | MT236901 | 100 | 100 | 5.15E-18 |
| OTU 169 | *Unknown* | *g__Malassezia* | OP699488 | 97.872 | 100 | 2.40E-16 |
| OTU 169 | *Unknown* | *g__Malassezia* | OL691887 | 97.872 | 100 | 2.40E-16 |
| OTU 175 | *Unknown* | *g__Clavispora* | AY321464 | 100 | 100 | 3.55E-32 |
| OTU 175 | *Unknown* | *g__Clavispora* | FJ183442 | 100 | 100 | 3.55E-32 |
| OTU 175 | *Unknown* | *g__Clavispora* | KP764997 | 100 | 100 | 3.55E-32 |
| OTU 184 | *Unknown* | *g__Penicillium* | AF033438 | 100 | 100 | 1.99E-83 |
| OTU 184 | *Unknown* | *g__Penicillium* | KX515667 | 100 | 100 | 1.99E-83 |
| OTU 184 | *Unknown* | *g__Penicillium* | ON712124 | 100 | 100 | 1.99E-83 |
| OTU 191 | *Unknown* | *g__Cladosporium* | EF679363 | 97.297 | 100 | 8.27E-67 |
| OTU 198 | *Unknown* | *g__Clavispora* | AY321464 | 100 | 100 | 3.55E-32 |
| OTU 198 | *Unknown* | *g__Clavispora* | FJ183442 | 100 | 100 | 3.55E-32 |
| OTU 198 | *Unknown* | *g__Clavispora* | KP764997 | 100 | 100 | 3.55E-32 |
| OTU 222 | *Unknown* | *g__Didymella* | MN973533 | 97.419 | 100 | 4.04E-70 |
| OTU 222 | *Unknown* | *g__Didymella* | MK836111 | 97.419 | 100 | 4.04E-70 |
| OTU 222 | *Unknown* | *g__Didymella* | FJ427003 | 97.419 | 100 | 4.04E-70 |
| OTU 226 | *Unknown* | *g__Aspergillus* | MH779840 | 100 | 100 | 4.84E-95 |
| OTU 233 | *Unknown* | *g__Toxicocladosporium* | OK664717 | 100 | 100 | 5.19E-10 |
| OTU 233 | *Unknown* | *g__Ascomycota_gen_Incertae_sedis* | MK405865 | 100 | 100 | 5.19E-10 |
| OTU 233 | *Unknown* | *g__Teratosphaeriaceae_gen_Incertae_sedis* | UDB05438955 | 100 | 100 | 5.19E-10 |
| OTU 266 | *Unknown* | *g__Sporobolomyces* | MK050408 | 98.544 | 100 | 1.48E-100 |
| OTU 266 | *Unknown* | *g__Sporobolomyces* | AY015438 | 98.544 | 100 | 5.32E-100 |
| OTU 275 | *Unknown* | *g__Fusarium* | KX281175 | 100 | 100 | 1.22E-85 |
| OTU 275 | *Unknown* | *g__Fungi_gen_Incertae_sedis* | KX343089 | 100 | 100 | 1.22E-85 |
| OTU 275 | *Unknown* | *g__Trichoderma* | AF443920 | 99.415 | 100 | 5.69E-84 |
| OTU 315 | *Unknown* | *g__Aspergillus* | EF652088 | 100 | 100 | 7.90E-93 |
| OTU 315 | *Unknown* | *g__Aspergillaceae_gen_Incertae_sedis* | JN839954 | 100 | 96 | 6.15E-89 |
| OTU 352 | *Unknown* | *g__Aspergillus* | KY087772 | 100 | 100 | 1.01E-91 |
| OTU 356 | *Unknown* | *g__Ramularia* | KX287521 | 99.296 | 100 | 1.69E-68 |
| OTU 356 | *Unknown* | *g__Ramularia* | KJ504766 | 98.592 | 100 | 7.87E-67 |
| OTU 356 | *Unknown* | *g__Ramularia* | KJ504792 | 98.592 | 100 | 7.87E-67 |
| OTU 404 | *Unknown* | *g__Alternaria* | KC584231 | 100 | 100 | 2.39E-77 |
| OTU 404 | *Unknown* | *g__Alternaria* | MW244999 | 100 | 100 | 2.39E-77 |
| OTU 404 | *Unknown* | *g__Alternaria* | KT268390 | 100 | 100 | 2.39E-77 |
| OTU 406 | *Unknown* | *g__Byssochlamys* | JN564480 | 100 | 100 | 7.51E-88 |
| OTU 406 | *Unknown* | *g__Paecilomyces* | PP101422 | 100 | 100 | 7.51E-88 |
| OTU 406 | *Unknown* | *g__Paecilomyces* | FJ389944 | 98.851 | 100 | 1.63E-84 |
| OTU 415 | *Unknown* | *g__Ogataea* | KY104446 | 100 | 100 | 1.81E-08 |
| OTU 415 | *Unknown* | *g__Kuraishia* | KM065941 | 100 | 100 | 1.81E-08 |
| OTU 442 | *Unknown* | *g__Paracamarosporium* | KU900326 | 100 | 100 | 1.09E-75 |
| OTU 442 | *Unknown* | *g__Paraconiothyrium* | KY979754 | 99.342 | 100 | 5.07E-74 |
| OTU 442 | *Unknown* | *g__Pseudocamarosporium* | MN562150 | 99.342 | 100 | 5.07E-74 |
| OTU 477 | *Unknown* | *g__Fusarium* | FJ645740 | 99.371 | 100 | 6.86E-78 |
| OTU 477 | *Unknown* | *g__Ascomycota_gen_Incertae_sedis* | KR709019 | 99.371 | 100 | 6.86E-78 |
| OTU 479 | *Unknown* | *g__Fungi_gen_Incertae_sedis* | KX515101 | 100 | 100 | 8.55E-77 |
| OTU 479 | *Unknown* | *g__Aureobasidium* | MW724138 | 98.649 | 96 | 4.00E-70 |
| OTU 483 | *Unknown* | *g__Fungi_gen_Incertae_sedis* | KX515092 | 100 | 100 | 1.71E-125 |
| OTU 483 | *Unknown* | *g__Filobasidium* | AF190007 | 99.587 | 100 | 7.96E-124 |
| OTU 483 | *Unknown* | *g__Fungi_gen_Incertae_sedis* | KX515095 | 99.587 | 100 | 7.96E-124 |
| OTU 484 | *Unknown* | *g__Pseudopithomyces* | MG828953 | 98.039 | 100 | 3.07E-71 |
| OTU 523 | *Unknown* | *g__Didymella* | MN973533 | 97.945 | 96 | 2.37E-67 |
| OTU 523 | *Unknown* | *g__Didymella* | MK836111 | 97.945 | 96 | 2.37E-67 |
| OTU 523 | *Unknown* | *g__Ascomycota_gen_Incertae_sedis* | MT236992 | 97.945 | 96 | 2.37E-67 |
| OTU 544 | *Unknown* | *g__Vishniacozyma* | DQ000318 | 98.571 | 100 | 1.00E-65 |
| OTU 724 | *Unknown* | *g__Vishniacozyma* | UDB0610977 | 98.026 | 100 | 3.94E-70 |

**a =** Genera assigned using the UNITE v8.2 database used in this study, **b =** Genera assigned using the latest (24-04-04) UNITE v10.0 database, **c =** QcovHSP stands for quer coverage per high scoring pair
